# Novel strategies to overcome tumor immunotherapy resistance using CAR NK cells

**DOI:** 10.3389/fimmu.2025.1550652

**Published:** 2025-05-29

**Authors:** Jun-Hui Guo, Ali Afzal, Sadia Ahmad, Ghazala Saeed, Amna Rehman, Umair Ali Khan Saddozai, Lei Liu, Shi-Hao Guo, Xin-Ying Ji, Muhammad Babar Khawar

**Affiliations:** ^1^ Department of Oncology, The Second Affiliated Hospital of Henan University of Traditional Chinese Medicine, Henan Province Hospital of Traditional Chinese Medicine, Zhengzhou, Henan, China; ^2^ Institute of Translational Medicine, Medical College, Yangzhou University, Yangzhou, China; ^3^ Jiangsu Key Laboratory of Experimental and Translational Non-Coding RNA Research Yangzhou, Yangzhou, China; ^4^ Institute of Zoology, University of the Punjab, Lahore, Pakistan; ^5^ Department of Cardiovascular Surgery, The Second Affiliated Hospital of Zhengzhou University, Zhengzhou, Henan, China; ^6^ Department of Colorectal Surgery, The First Affiliated Hospital, Zhengzhou University, Zhengzhou, Henan, China; ^7^ Center for Molecular Medicine, Faculty of Basic Medical Subjects, Shu-Qing Medical College of Zhengzhou, Zhengzhou, Henan, China; ^8^ Department of Nuclear Medicine, Henan International Joint Laboratory for Nuclear Protein Regulation, the First Affiliated Hospital, Henan University College of Medicine, Kaifeng, Henan, China; ^9^ Applied Molecular Biology and Biomedicine Lab, Department of Zoology, University of Narowal, Narowal, Pakistan

**Keywords:** CAR NK cells, immunotherapy resistance, tumor microenvironment, NK cell homing, cancer immunotherapy

## Abstract

While immunotherapy faces obstacles, the emergence of chimeric antigen receptor (CAR) engineered natural killer (NK) cells is paving new ways and might become a preferred option over CAR T cells very soon. CAR NK introduce diverse cytotoxic mechanisms offering novel strategies to combat tumor immunotherapy resistance. Concurrently, improvements in NK cell homing and gene-edited CAR NK cell therapy offer promising avenues for overcoming challenges in cancer immunotherapy. Our review addresses resistance mechanisms and engineering strategies to enhance CAR NK cell functionality by improving NK cell homing and migration to tumor sites, emphasizing insights from preclinical and clinical studies.

## 1 Introduction

**Figure 1:**
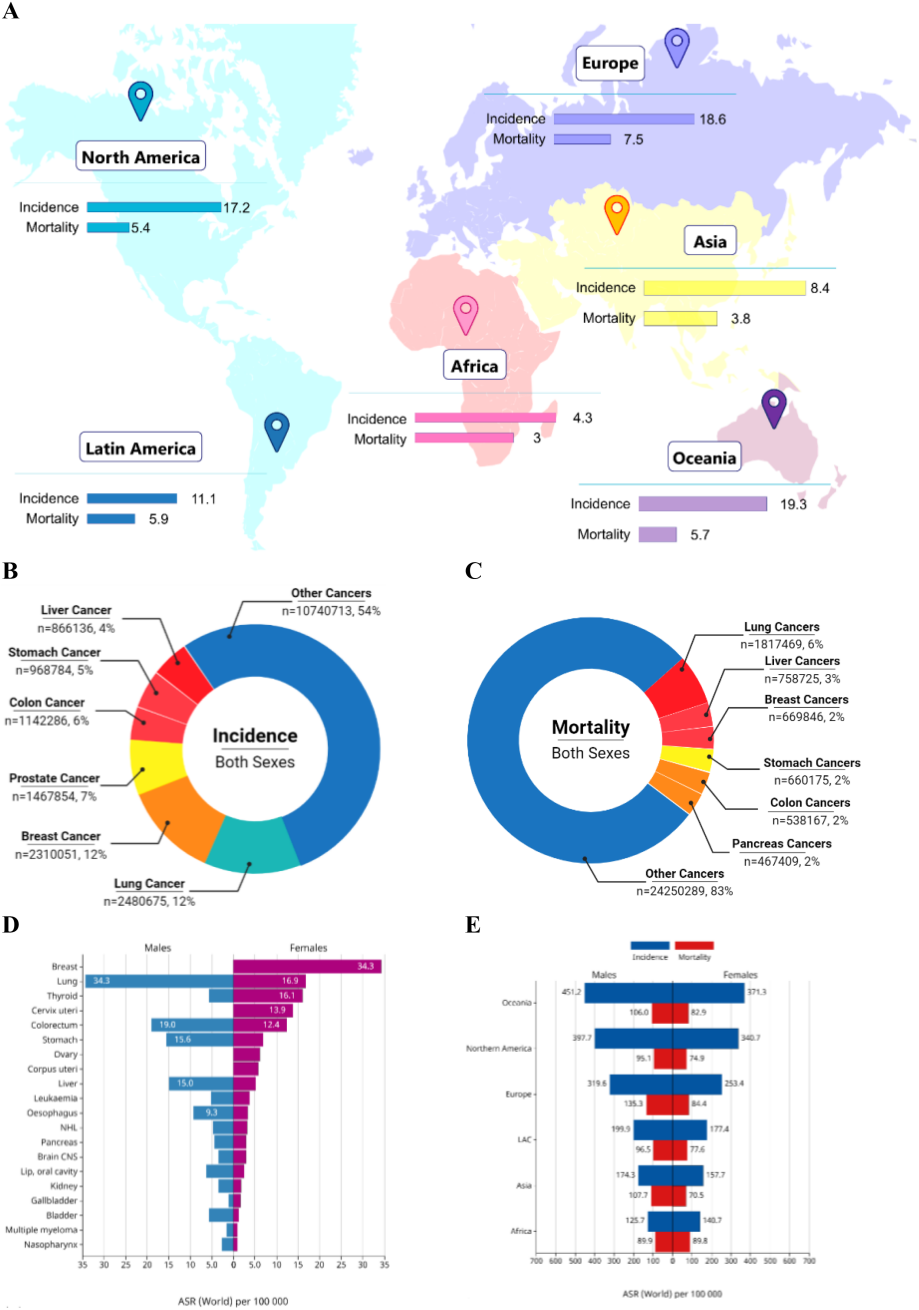
Summary of global cancer burden. **(A)** World map illustrating the age-standardized incidence and mortality rates of cancers across continents. **(B)** Pie chart showing the global distribution of cancer incidence among both sexes for the top cancer types. **(C)** Pie chart showing the global distribution of cancer-related mortality among both sexes for the top cancer types. **(D)** Bar chart comparing age-standardized incidence rates (ASR) between males and females across major cancer types. **(E)** Stacked bar chart depicting combined incidence and mortality rates for males and females across all continents. The data was retrieved from GLOBOCAN Cancer Today [Adapted and modified from (Afzal, Abbasi et al. 2)].

Irrespective of major developments in treatment, cancer persists as a leading cause of global morbidity and mortality. In 2025, the United States is expected to report approximately 2.0 million new cancer cases—1.05 million in males and 0.99 million in females—and an estimated 0.6 million cancer-related deaths, with 0.3 million among males and 0.29 million among females (1). A summary of global statistics for the year 2022 is depicted in **Figure 1**. Despite advances in conventional treatments such as surgery, chemotherapy, and radiation, many cancers, particularly solid tumors, demonstrate resistance, relapse, or metastasis. Current therapeutic options often fail to achieve durable responses in advanced-stage cancers which highlight the urgent need for more effective strategies that can improve patient outcomes while minimizing toxicity.

Currently, immunotherapy has emerged as a promising strategy which offers more durable responses compared to conventional treatments such as radiotherapy and chemotherapy (3). It is now considered the “fifth pillar” of cancer treatment, alongside chemotherapy, targeted therapy, surgery, and radiotherapy (4). Cancer immunotherapy utilizes components of the immune system to protect the host from primary tumor development or tumor escape, processes that are strictly regulated by immune checkpoints—cell surface receptors that modulate the activation or inhibition of immune responses (5).

Recent advances in immunotherapy, such as immune checkpoint inhibitors (6), tumor vaccines (7), and adoptive cell therapies (ACT) (8), have significantly transformed the cancer treatment landscape by harnessing the immune system’s ability to specifically target and eliminate cancer cells. These approaches offer durable responses and new hope for patients with cancers that were previously considered untreatable. Modern therapies include chimeric antigen receptor (CAR) T cell therapy, checkpoint blockade therapy, and ACT (9, 10).

CAR-engineered immune cells represent a promising avenue in tumor immunotherapy (11). CARs are synthetic receptors genetically introduced into immune cells and comprise several key components: (1) a single-chain variable fragment (scFv) extracellular antigen-binding domain for recognizing target antigens, (2) a hinge region for flexibility and spatial orientation, (3) a transmembrane domain for anchoring, (4) intracellular signaling domains comprising a primary activation domain (typically derived from CD3ζ of the T cell receptor), and (5) one or more costimulatory domains (such as CD28, 4-1BB, or OX40) that enhance T cell activation, proliferation, and persistence (12).

Importantly, CAR T cell therapy is a specialized form of ACT, wherein T cells are either naturally selected or genetically engineered before reinfusion into patients. Other ACT strategies include TCR-engineered T cell therapies and CAR NK cell therapies. In addition to ACT, cancer immunotherapy strategies also involve peptide-based cancer vaccines (7), oncolytic viruses (13), antigen-presenting cell (APC) therapies (14), tumor-infiltrating lymphocyte therapy (15), neoantigen-based therapies (16), and immune checkpoint blockade (17).

CAR T cell therapy has demonstrated remarkable outcomes in individuals diagnosed with B cell acute lymphoblastic leukemia and other hematologic malignancies (18). Beyond leukemia, it has shown promising therapeutic potential in various other hematologic cancers such as diffuse large B-cell lymphoma, chronic lymphocytic leukemia, and multiple myeloma (19). Furthermore, ongoing advancements are extending its application to solid tumors including glioblastoma, breast, and pancreatic cancers, although clinical responses remain variable due to the complex tumor microenvironment (TME) (20). However, its efficacy against solid tumors remains limited (21). Challenges associated with ACT include immune-related adverse events such as off-target effects, on-target/off-tumor toxicity, cytokine release syndrome (CRS), and suboptimal *in vivo* persistence of engineered cells (22). Moreover, the immunosuppressive TME, inadequate generation and function of tumor-specific CD8+ T cells, limited neoantigen availability due to impaired antigen processing and presentation, and epigenetic alterations further hinder effective immune responses (23, 24). Immune-related adverse events, characterized by inflammatory responses against healthy tissues, continue to pose significant challenges to the broader application of immunotherapies (5).

In addition to T cells, natural killer (NK) cells have gained attention in ACT. CAR-engineered NK cells offer distinct advantages due to their innate cytotoxic capabilities and lower risk of causing severe toxicities. NK cells, a type of innate lymphoid cell, play crucial roles in immune surveillance against solid tumors, hematologic malignancies, and metastatic dissemination (25). While there is growing interest in NK cell-based therapies (26), a recurring challenge is the lack of standardized definitions and assays for assessing NK cell cytotoxicity (27), which complicates comparative analyses across studies.

We previously reviewed the development of CAR NK cells, highlighting their evolution from natural cytotoxicity to engineered designs aimed at enhancing antitumor activity (23, 28–30). CAR NK cells offer multiple advantages: They are less likely to cause CRS (31), exhibit higher safety profiles through multiple killing mechanisms (32), and are more cost-effective than CAR T cell therapies (33). Consequently, they are more accessible to patients and show promise in treating solid tumors such as glioblastoma, a setting where CAR T cell therapies have struggled (34, 35). In this review, we explore the emerging frontiers of tumor immunotherapy, focusing on the efficacy, resistance mechanisms, and strategic innovations surrounding CAR NK cell therapy to advance future clinical applications.

## 2 Natural killer cells and their therapeutic potential

NK cells were first identified by Rolf Kiessling and colleagues in the early 1970s, who observed spontaneous cytotoxic activity of lymphocytes against tumor cells in murine models (36, 37). Around the same time, similar cytotoxic activity was reported in human peripheral blood lymphocytes by Jondal and Pross (38), which established the concept of ‘natural’ cytotoxicity. These discoveries laid the foundation for the characterization of NK cells as a unique subset of lymphocytes, further described by Herberman in 1976 (39, 40).

NK cells (CD56^+^CD3^-^) exhibit innate cytotoxicity and immunoregulatory functions. They inhibit tumor initiation, proliferation, and metastasis through mechanisms resembling CD8^+^ cytotoxic T cells but operate independently of somatically rearranged, antigen-specific T cell receptors (TCRs) (41, 42). NK cells also produce cytokines, chemokines, and growth factors that orchestrate adaptive immune responses (43). They can be derived from peripheral blood (PB), umbilical cord blood (UCB), hematopoietic stem cells, and human pluripotent stem cells (hPSCs) (44).

NK cells, classified as group 1 innate lymphoid cells (ILC1s), share developmental pathways with T and B cells. Their differentiation involves the migration of CD34^+^CD45RA^+^ hematopoietic progenitor cells from the bone marrow to various anatomical sites such as the uterus, tonsils, liver, and spleen, where under the influence of IL-15, they mature into NK cells (45). The exact developmental trajectory—whether linear or non-linear—remains under investigation.

Unlike T and B cells, NK cells lack somatically recombined antigen receptors. Their function is regulated by a dynamic balance between activating receptors (*e.g.*, DNAM-1, NKp44, NKp30, NKp46, and NKG2D) and inhibitory receptors (*e.g.*, NKG2A for non-classical HLA-I molecules and killer immunoglobulin-like receptors, KIRs, for classical HLA-I molecules) (26, 43, 45–48). Tumor cells, often downregulating HLA molecules, are particularly susceptible to NK cell-mediated killing via the “missing-self” recognition mechanism (49). Upon sensing stress-induced ligands, NK cells eliminate malignant cells through direct cytotoxicity, including granule-mediated apoptosis and antibody-dependent cellular cytotoxicity (ADCC), or by secreting pro-inflammatory cytokines—a process termed the “induced self” mechanism (50, 51). Inhibitory signals, conversely, ensure self-tolerance and prevent unwanted NK cell activation against healthy tissues (26, 43, 45, 48).

NK cell infiltration within tumors correlates with improved outcomes across various cancers, such as liver (52), renal (53), melanoma (54), breast (55), and lung cancers (56).

Importantly, NK cells are considered ideal candidates for adoptive immunotherapy because they confer graft-versus-tumor effects without inducing graft-versus-host disease (GvHD). Furthermore, they are less likely to cause long-term adverse effects like malignant transformation or autoimmunity due to their low cytokine toxicity and short lifespan (57).

## 3 CAR NK cell therapy

Radiotherapy induces DNA damage, which upregulates the expression of NKG2D ligands on cancer cells (41). This promotes NK cell activation and enhances cytotoxicity against cancer cells. Combining local radiotherapy with CAR NK cells may offer an alternative therapeutic strategy, particularly for solid tumors. A preclinical study using CAR NK cells combined with anti-PD-1 antibodies showed enhanced cytotoxic activity in both hematologic and solid tumors (58). While the PD-1 deletion arm is still being tested, the dual therapy led to significant tumor volume reduction in xenograft models. Furthermore, PD-1 knockout CAR NK cells have been shown to improve immune cell infiltration and increase tumor cell killing in preclinical ovarian cancer models. These modified NK cells effectively suppressed tumor growth while minimizing systemic toxicity (59).

ICB using antibodies such as anti-CTLA-4 or anti-PD-1/PD-L1 has shown effective results in various solid tumors and hematologic malignancies (60). NK cells, like T cells, express immune checkpoint molecules such as TIM-3, CTLA-4, PD-1, and LAG-3, which impair their anticancer activity (41). NK cell activity can be enhanced through checkpoint blockade (41). Therefore, combining CAR NK cells with checkpoint molecule deletion via gene editing, systemic checkpoint blockade, or co-expression of checkpoint inhibitors presents a promising strategy for patients with solid tumors.

Several clinical trials are optimizing the efficacy of checkpoint inhibitors in combination with NK cells. For instance, the current management of Merkel cell carcinoma involves avelumab combined with the IL-15 superagonist N-803, which targets CD16 on haNK cells. This approach, evaluated after checkpoint inhibitor therapy, demonstrated an objective response rate of 36.4%, a median response duration of 18.9 months, and 12- and 24-month overall survival rates of 88.2% and 70.8%, respectively, despite a 30% incidence of grade 3–5 treatment-related adverse events (61). For gemcitabine-refractory biliary tract cancer, pembrolizumab (Keytruda) with allogeneic NK cells (“SMT-NK”) is under evaluation (NCT03937895). Furthermore, a phase I/II study (NCT04143711) is assessing the tolerability, pharmacokinetics, and clinical functionality of DF1001 combined with pembrolizumab in patients with metastatic solid tumors.

Another approach to enhance CAR NK cell therapy involves using antibodies to target a broad range of tumor-associated antigens (TAAs), enabling NK cells to induce tumor necrosis through ADCC (62). Administration of anti-CD20 or anti-GD2 antibodies followed by infusion of allogeneic NK cells in patients with refractory non-Hodgkin lymphoma or neuroblastoma has shown promising outcomes (63). However, activation of target cells and cytokines often downregulates CD16 expression on NK cells, impairing ADCC (64). Genetic engineering of NK cells to express a non-cleavable form of CD16 addresses this limitation (30).

FT596, a genetically modified NK cell product, overcomes tumor resistance associated with CD19 antigen loss when combined with therapeutic antibodies by exhibiting multi-antigen targeting activity. Based on promising preclinical results, FT596 is being evaluated in a phase I clinical study (NCT04245722). Sequential administration of “off-the-shelf” CAR NK cells followed by CAR T cells may offer synergistic tumor killing, durable anticancer efficacy, and reduced risks of neurotoxicity and CRS-associated with CAR T cell therapy.

## 4 CAR NK *vs* CAR T therapy

Immunotherapy—including checkpoint inhibitors, cytokines, antibody therapies, and CAR T cells—has greatly advanced cancer treatment, especially for blood cancers. CAR T cells revolutionized tumor therapy with durable clinical responses over the past 26 years (46). However, several limitations hinder their broader applicability, comprising CRS (65), immune effector cell-associated neurotoxicity syndrome (66), loss of T cell functionality and viability (67), and antigen escape due to tumor heterogeneity (68). Moreover, the manufacture of autologous T lymphocytes, including CAR T and TCR-engineered T cells (TCR-T), is technically demanding and logistically challenging for personalized therapies (69). Thus, a strong demand has emerged for novel cellular therapies such as CAR NK cells, which promise superior safety, scalability, and efficacy.

CAR NK cells when compared with CAR T cells (**Table 1**), address many of these shortcomings. They are associated with reduced risks of neurotoxicity, CRS, and tumor lysis syndrome, and exhibit multifaceted cytotoxic mechanisms (28, 78). Additionally, CAR NK cells can be feasibly manufactured as “off-the-shelf” products, offer enhanced infiltration into solid TMEs, overcome TME-mediated immunosuppression, and can target a broader range of tumor-associated antigens, thereby minimizing relapse risks (41, 79).

**Table 1:** Key differences between CAR T and CAR NK cells.

Feature	CAR NK Cells	CAR T Cells	References
Mechanism of Action	Innate immune response; does not require antigen presentation	Adaptive immune response; recognizes antigens independently of MHC presentation	(70)
Cytotoxic Mechanisms	Multiple mechanisms: FAS-FASL-mediated apoptosis, TRAIL-mediated apoptosis, ADCC, granule-mediated lysis (perforin & granzyme)	Predominantly CAR-mediated direct cytotoxicity and cytokine release	(71)
Side Effects	Lower risk of CRS and GvHD	Higher risk of CRS and neurotoxicity	(72)
Persistence in Patients	Limited *in vivo* persistence; may require repeated administration	Better *in vivo* persistence; long-term survival of CAR T cells	(73)
Manufacturing	Easier and faster; can be produced as an allogeneic therapy	Complex and time-consuming; autologous manufacturing required	(74)
Cost and Accessibility	More cost-effective and widely accessible	Expensive and less accessible	(75)
Effectiveness in Hematologic Malignancies	Effective; ongoing clinical trials are promising	Proven efficacy in B cell malignancies, *e.g.*, leukemia and lymphoma	(73, 76)
Immunosuppressive TME	Better adaptation and resistance to immunosuppressive TME	Prone to inhibition by immunosuppressive TME	(77)

ADCC, Antibody-dependent cellular cytotoxicity; CRS, Cytokine release syndrome; FAS, First apoptosis signal receptor; FASL, First apoptosis signal receptor ligand; TME, Tumor microenvironment; GvHD, graft-versus-host disease; TCR, T cell receptor; TRAIL, Tumor necrosis factor-related apoptosis-inducing ligand.

Various sources are being explored for NK cell-based therapies, including autologous NK cells, allogeneic NK cells, genetically engineered NK cells, and NK cell lines derived from PB or stem cells (80). There are two major types of NK cell therapies: Autologous and allogeneic, each offering distinct advantages depending on the clinical scenario. By learning from the limitations of both CAR T and unmodified NK cell therapies, CAR NK cells are increasingly recognized as an innovative and powerful approach to improve remission rates and reduce tumor recurrence (22, 81).

## 5 Mechanisms of resistance to tumor immunotherapy

Beyond tumor cells, the TME consists of a complex network of immune and stromal components that significantly influence cancer progression and response to therapy. Among these, tumor-infiltrating lymphocytes (TILs) play a crucial role in antitumor immune responses. TILs include both regulatory T cells (Tregs), which suppress immune activity and contribute to tumor immune evasion, and effector T cells (Teffs), which mediate cytotoxic responses against tumor cells (82, 83). Other key elements of the TME include dendritic cells, fibroblasts, stromal cells, bone marrow-derived inflammatory cells, vascular endothelial cells, and extracellular matrix (ECM) components, along with regulatory molecules such as cytokines, hormones, and reactive oxygen species (84).

The patient’s response to immunomodulatory agents is largely shaped by dynamic interactions between tumor cells and the TME. Tumors are often classified as either “hot” or “cold” based on TIL infiltration. Hot tumors, with abundant TILs, are typically more responsive to immunotherapy due to heightened immune activation (85). In contrast, cold tumors, characterized by low TIL infiltration, frequently exhibit immune exclusion or suppression, leading to resistance against immunotherapeutic interventions (86). Factors such as paracrine signaling from fibroblasts, the immunosuppressive activity of tumor-associated macrophages, and the cytotoxic potential of TILs critically influence tumor progression and immune evasion within the TME (87).

**Figure 2:**
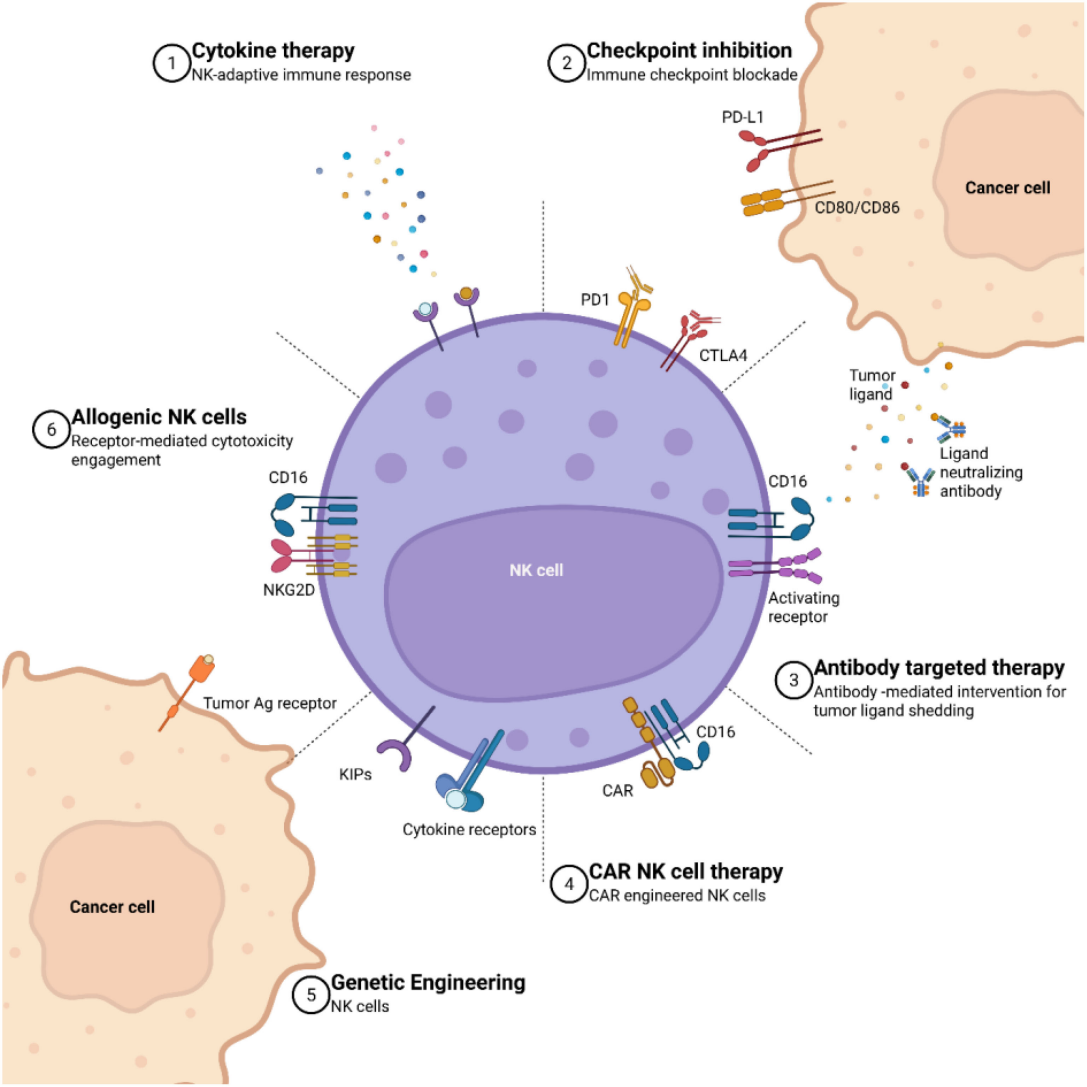
A summary of various therapeutic interactions in CAR NK therapy. (1) Cytokine therapy is represented as the stimulation of NK cells and subsequent activation of the adaptive immune response through the administration of cytokines. (2) Immune checkpoint inhibition showcases the blockade of inhibitory checkpoints such as PD1 and CTLA4 on NK cells, PDL1 and CD80/CD8 on cancer cells, aiming to enhance the immune response against tumors. (3) Antibody-targeted therapy is an interaction between CD16 and activating receptors on NK cells with tumor ligands, while cancer cells release tumor ligands and ligand-neutralizing antibodies to modulate this process. (4) The expression of CD16 on CAR NK cells highlight the use of synthetic receptor systems to improve the targeting specificity and cytotoxic potential of NK cells against tumor cells. (5) Genetically engineered CAR NK cells with the incorporation of kinase inhibitory domains (KIPs) and cytokine receptors, showcase advanced genetic modifications aimed at improving the efficacy and persistence of CAR NK cells in cancer therapy. (6) Additionally, allogeneic NK features the interaction between the tumor antigen receptor on cancer cells and NK cell receptors like NKG2D and CD6, demonstrating the potential of allogeneic NK cells to recognize and target cancer cells (Adapted from (89) Licensed under CC BY.).

Resistance to immunotherapy driven by the TME is multifactorial, involving both intrinsic properties of tumor cells and extrinsic environmental influences (88), as depicted in **Figure 2**. Two major contributors to this resistance are tumor cell heterogeneity and tumor escape mechanisms (30). Specifically, alterations within the TME—such as immune exclusion in cold tumors and the activation of suppressive signaling pathways—constitute key extrinsic mechanisms undermining antitumor immunity (82).

### 5.1 Tumor cell heterogeneity

Tumor cell heterogeneity significantly impacts the response to immunotherapy and manifests as both intratumoral and intertumoral variability which affects immune cell infiltration and the broader TME (90).

Intratumoral heterogeneity refers to genetic and cellular diversity within a single tumor. High levels of intratumoral heterogeneity are associated with poor T cell infiltration and resistance to immune checkpoint blockade (ICB) therapies (91). Tumor cells can also form spatially organized immunosuppressive niches, particularly in regions with sparse T cell presence and a high concentration of suppressive macrophages (92). Additionally, during the development of resistance to ICBs, the expression of PD-1 and its ligands (PD-L1 and PD-L2) is upregulated by interferon-γ which enhances the production of chemokines like CXCL9 and CXCL10. This leads to a pro-apoptotic environment that adversely affects immune cell infiltration (83). Within the TME, molecules such as CX3CL1 further promote the accumulation of immunosuppressive immune cells (93). Moreover, heterogeneous expression and downregulation of MHC-I in some tumor cells impair CD8+ T cell-mediated responses, reducing the efficacy of immunotherapy (94).

Genetic diversity within the TME can also cause variable responses to immunotherapy. Clonal subpopulations within heterogeneous tumors often exhibit distinct growth patterns and unique immune escape mechanisms (95). Immunosuppressive tumor cell populations, by remodeling the TME, further contribute to immune evasion and therapeutic resistance (91).

Although tumor heterogeneity presents substantial challenges for immunotherapy, it also offers opportunities for developing targeted interventions. A deeper understanding of the mechanisms driving tumor heterogeneity is crucial for enhancing the effectiveness of future therapeutic strategies.

### 5.2 Tumor escape mechanisms

Tumor escape from immune surveillance involves multiple strategies that inhibit NK cell function and adaptive immune responses. Immunosuppressive factors within the TME interfere with NK cell interactions and downregulate activating receptors, while tumor cells may also trigger inhibitory NK receptors to evade immune attack (44).

The TME contains various immunosuppressive cells that, under physiological conditions, maintain self-tolerance and prevent autoimmunity, but in cancer, contribute to immune evasion. Tregs, for example, suppress effector T cells (Teffs) through multiple mechanisms: Inhibiting the maturation of APCs and MHC expression, secreting inhibitory cytokines like IL-35, IL-10, and TGF-β to limit T cell proliferation and activation, and directly killing APCs and T cells via perforin and granzyme (82).

Adaptive immune resistance further enables tumors to escape immunotherapy. In this process, T cell attacks induce IFN-γ production, which is followed by the upregulation of immune checkpoint molecules such as B7-H1 (PD-L1) within the TME, promoting local immune evasion (88). Efforts to overcome immune escape are ongoing, including the development of CAR NK cells through genetic engineering, aimed at enhancing the antitumor properties of NK cells and preventing immune evasion.

## 6 Strategies to overcome tumor immunotherapy resistance

Tumor resistance to immunotherapy remains a major obstacle in cancer treatment. It can be broadly categorized into primary resistance, where tumors inherently evade immune attack, and acquired resistance, where tumors develop escape mechanisms over time (96). Resistance mechanisms include loss of antigen presentation, upregulation of immunosuppressive pathways, and the establishment of an immunosuppressive TME (97).

Researchers are investigating combination strategies to enhance the efficacy of CAR NK cells. Unlike CAR T cells, CAR NK cells possess natural cytotoxicity, a lower risk of CRS and GvHD, and the ability to target tumors through multiple mechanisms (28). Despite these advantages, CAR NK cells still face challenges such as hostile TMEs, limited persistence, and tumor immune evasion (77).

This section discusses key strategies to improve CAR NK cell therapy. These include combination approaches integrating chemotherapy, radiotherapy (98), and ICB (99) to modulate the TME and enhance tumor targeting. Preclinical models and early clinical data have demonstrated that radiotherapy can enhance NK cell infiltration and antigen presentation, thereby overcoming immunotherapy resistance in tumors like small cell lung cancer (100). Similarly, the application of ICB in tandem with CAR NK cells reinvigorates exhausted NK cells and boosts their persistence in immune-suppressive TMEs, particularly in metastatic breast and lung cancers (30).

Furthermore, antibody-dependent cellular cytotoxicity (ADCC), targeting of novel tumor antigens, and advanced CAR designs—such as those encoding cytokines or checkpoint-modulating domains—have been shown to enhance NK cell cytotoxicity and survival (101). These strategies have already shown efficacy in hematologic malignancies, and emerging trials suggest applicability to solid tumors. Collectively, these combinatorial and next-generation approaches hold substantial promise in addressing tumor heterogeneity, immune evasion, and resistance mechanisms, thereby broadening the clinical success of CAR NK cell therapies.

### 6.1 Combination therapies

Single-agent immunotherapies demonstrate potential efficacy but often result in relatively low response rates. In contrast, combination therapies with targeted agents, chemotherapy, and radiotherapy achieve higher effectiveness. It is important to assess how chemotherapy or radiation can enhance antigen presentation, promote inflammation within the TME, and facilitate NK cell infiltration.

### 6.2 Case applications

Doxorubicin, a topoisomerase II inhibitor, induces immunogenic cell death, releasing tumor-associated antigens and damage-associated molecular patterns that activate immune responses (102). Chemotherapy enhances antitumor immunity by increasing tumor-specific antigens, promoting dendritic cell maturation, and improving CD8+ T cell activation through efficient antigen presentation (103, 104).

Chemotherapy can also promote inflammation within the TME, potentially contributing to tumor progression. This inflammation results from complex interactions involving signaling pathways and inflammatory mediators that reshape TME dynamics. Pro-inflammatory cytokines such as IL-1β and TNF-α are stimulated through pathways like TLR4 signaling and inflammasome activation (105). Chemotherapy can trigger a senescence-associated secretory phenotype, releasing inflammatory factors that support tumor growth and metastasis (105). Moreover, tumor-associated macrophages, particularly the M2 phenotype, are recruited and polarized, promoting immunosuppression and chemoresistance (106). Elevated inflammation, driven by enzymes like COX-2, can induce epithelial-to-mesenchymal transition, enhancing cancer cell invasion and metastasis (107). Prolonged inflammation establishes a supportive environment for tumor survival and relapse, complicating treatment outcomes (105).

Interestingly, chemotherapy enhances NK cell infiltration into tumors by modulating the TME and immune responses. Immunogenic cell death triggered by chemotherapy upregulates NK cell-activating ligands on cancer cells, facilitating their recognition and destruction (108). Chemotherapy also promotes the release of cytokines such as IL-2 and IFN-γ, which activate NK cells and support their migration into tumors (109). Certain chemotherapeutic agents restore NK cell functionality by increasing tumor cell susceptibility to NK cell-mediated killing (110).

Clinically, these mechanisms translate into improved outcomes. For instance, in breast cancer patients receiving neoadjuvant chemotherapy, heightened peripheral NK cell activity correlates with lymph node metastasis eradication (111). In metastatic melanoma, chemotherapy has been shown to restore NK cell functionality, potentially synergizing with immunotherapies (110). However, variability in patient responses underscores the need for personalized treatment strategies to optimize therapeutic success.

Immunotherapeutic agents combined with other anticancer therapies demonstrate synergistic effects due to differences in timing, localization, and complementary targeting of distinct immune checkpoint pathways. Chemoradiotherapy, for instance, enhances cancer cell immunogenicity through rapid release of tumor-specific antigens, promoting antigen presentation, inflammation within the TME, lymphocyte infiltration, and tumor lysis. Additionally, combination therapy modulates gene expression linked to tumor cell proliferation and chemokine production (46, 47, 112).

**Figure 3:**
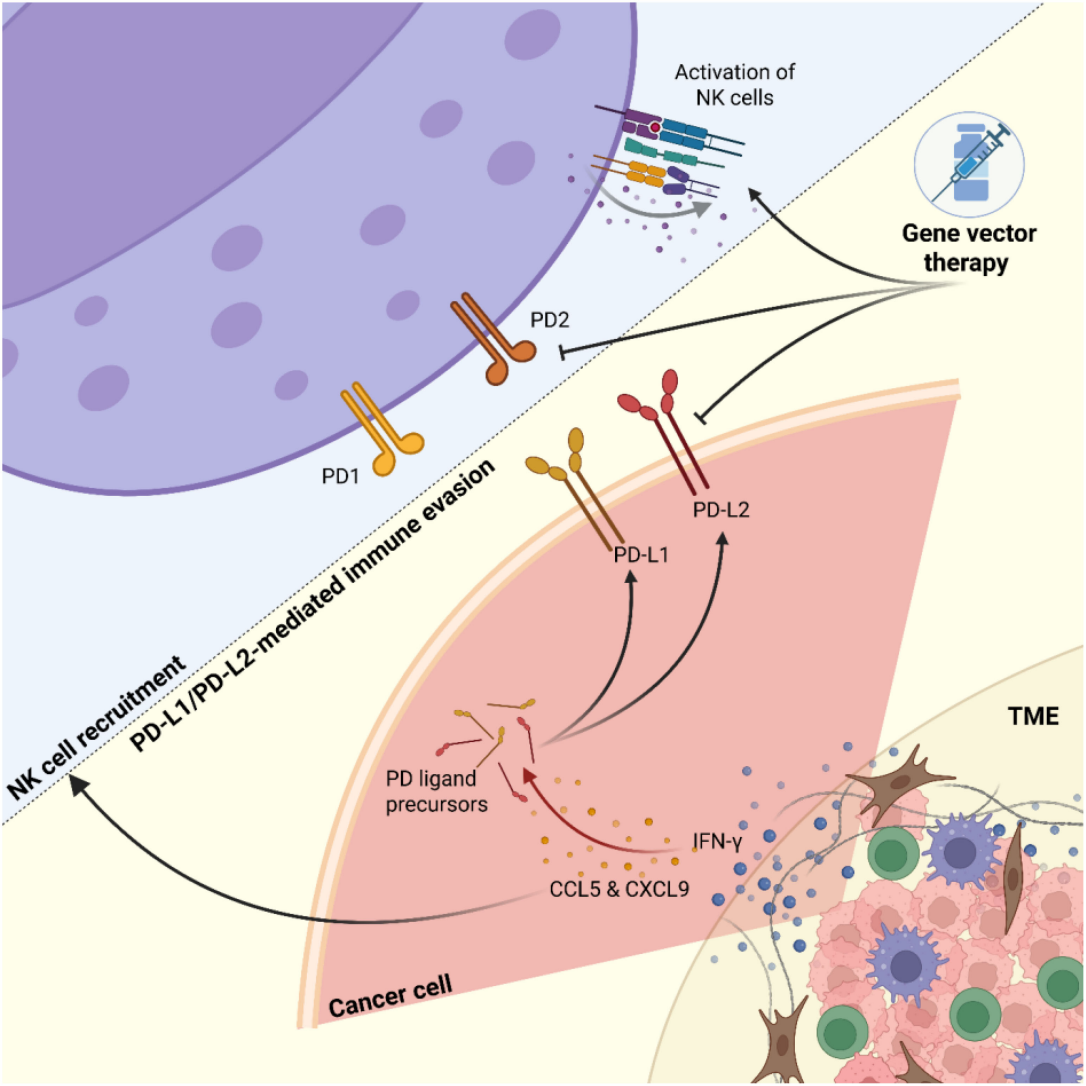
Dynamic interactions between tumors and NK cells. Tumor cells exhibit heterogeneity influenced by interferon-γ (IFN-γ) signaling, leading to increased chemokine secretion (e.g., CCL5, CXCL9) and upregulation of immune checkpoint molecules PD-L1 and PD-L2. Natural killer (NK) cells are recruited toward the tumor mass but face inhibition through PD-L1/PD-L2-mediated immune evasion. Gene vector therapy targets both tumor and NK cells to downregulate checkpoint molecule expression and upregulate NK cell-activating receptors, aiming to enhance anti-tumor immunity.

In small cell lung cancer immunotherapy, combining anti-CTLA agents with anti-PD-1 or anti-PD-L1 drugs shows potential synergistic effects. In breast cancer, Akt-targeted chemotherapy combined with anti-PD-1 immunotherapy demonstrates promising results in suppressing metastasis (46, 47, 112) (**Figure 3**).

Lymphodepleting chemotherapy, most commonly involving fludarabine and cyclophosphamide, is regularly administered before introducing allogeneic NK cells to preferentially eliminate lymphoid cells and prevent NK cell rejection by the recipient’s immune system (41). Lymphodepleting chemotherapy also helps reprogram the immunosuppressive TME by depleting regulatory T cells and myeloid-derived suppressor cells, generating a cytokine milieu that supports NK cell expansion and antitumor activity (41, 113). Intensive chemotherapy can further reduce tumor burden and improve the effector-to-target ratio before NK cell infusion (41).

For CAR NK cell therapy, chemotherapy can serve as an adjuvant therapeutic approach. Radiation therapy remains a prevalent palliative and curative treatment for various cancers. Stereotactic body radiotherapy (SBRT) has been shown to synergize with immunotherapies, including anti-CTLA4 and anti-PD-1 antibodies (114). Preclinical studies propose several mechanisms underlying this synergy. Radiation releases danger signals and immunostimulatory cytokines, increases tumor antigen availability, and destroys stromal cells that support cancer growth (115). These changes promote the recruitment and activation of APCs, subsequently stimulating a tumor-specific immune response (116).

Combination strategies integrating chemotherapy, radiotherapy, and immunotherapy can enhance CAR NK cell efficacy via remodeling the TME, promoting immune infiltration, and restoring NK cell functionality. However, optimizing dosing schedules, minimizing pro-tumor inflammation, and improving NK cell persistence remain critical avenues for future exploration.

### 6.3 Targeting novel antigens

CAR NK cells exhibit cytotoxic effects through both CAR-independent and CAR-dependent mechanisms. It is possible to design CAR constructs without costimulatory domains. CAR NK cells mainly exert antitumor responses through their inherent cytotoxicity, with direct CAR-mediated killing playing a secondary role. NK cells can be genetically modified to express non-signaling CARs, which enhance homing and adhesion to target cells without initiating direct killing signals. This modification enables selective cytotoxicity against tumor cells while minimizing damage to normal cells.

A variety of antigens with high expression on tumors and low expression on normal tissues have been identified as targets for CAR NK cell therapy, including mesothelin, HER2, CD19, and EGFR (117). Multiple clinical trials are underway targeting antigens such as PSMA, EpCAM, EGFR, BCMA, CD7, CD33, CD19, CD138, CD22, CD276, CS1, FLT3, IL13Rα, HER2, and Mucin-1 across various hematologic malignancies and solid tumors (43, 118). CD19 remains the most common target for B cell malignancies, while HER2 is frequently targeted in solid tumors like breast cancer.

Clinical trials are planned for EGFR- and IL13Rα-targeted CAR NK cell therapy in glioblastoma. HER2-targeted CAR NK cell therapy for locally progressive solid tumors is under recruitment (NCT04050709). Similarly, PSMA-targeted CAR NK cell therapy for prostate cancer is being evaluated (NCT03692663). Mesothelin, highly expressed in lung cancer and several other solid tumors, is also considered a potential target (118) as shown in **Table 2**.

**Table 2:** The proposed mechanisms of action for the treatment.

Treatment	Description	Mechanisms	References
Combination Therapies with Immunotherapy	Synergistic effects are observed due to differences in timing, location, and non-overlapping, as well as complementary effects of immune checkpoint pathways.	Enhancement of cancer cell immunogenicity, rapid release of TAAs, increased antigen presentation, lymphocyte infiltration, induction of tumor lysis, changes in gene expression, and chemokine expression.	(46, 47)
Immunotherapy for Small Cell Lung Cancer	Anti-CTLA agents and anti-PD-1 or anti-PD-L1 drugs are combined to show potential synergistic effects.	Enhanced immunotherapeutic efficacy against Small Cell Lung Cancer.	(112)
Lymphodepleting Chemotherapy	Depletion of Tregs and MDSCs may reprogram the immunosuppressive TME and enhance NK cell expansion. Intensive chemotherapy can be used to reduce tumor burden before NK cell infusion.	Improved NK cell function, expansion, and reduced tumor burden.	(113)
Stereotactic body radiotherapy (SBRT) and Immunotherapy	SBRT shows synergistic efficacy with immunotherapy (anti-CTLA4 antibody and anti-PD1). DNA damage from radiotherapy upregulates NKG2D ligands on cancer cells, enhancing NK cell activation.	Enhanced cancer-specific immune responses, increased cytotoxicity against cancer cells, and the abscopal effect.	(60)
ICB	CAR NK cells with checkpoint molecule deletions or co-expression of ICB molecules show promise for solid tumors.	Enhanced anticancer effects by blocking immune checkpoint molecules.	(60)
CAR NK Cell Therapy with Antibodies	Downregulation of CD16 expression on NK cells is addressed by genetic engineering techniques, leading to consistent CD16 expression. FT596 is a promising CAR NK cell product with multi-antigen targeting.	Enhanced tumor cell killing and overcoming resistance to therapeutic antibodies.	(63)
Progressive CAR NK Cell Therapy and CAR T	Administering “off-the-shelf” CAR NK cells before CAR T cells promotes rapid and durable anticancer efficacy with reduced risk of neurotoxicity and CRS.	Enhanced tumor reduction and safety profile.	(113)
Combining Checkpoint Inhibitors with NK Cells	Clinical trials are testing checkpoint inhibitors with NK cells for cancer treatment, including Merkel cell carcinoma and gemcitabine-refractory biliary tract cancer.	Evaluating the efficacy and safety of combining checkpoint inhibitors with NK cells.	(46)

CAR, Chimeric Antigen Receptor; CD16, Cluster of Differentiation 16; CRS, Cytokine Release Syndrome; CTLA, Cytotoxic T-Lymphocyte-Associated Protein 4; ICB, Immune Checkpoint Blockade; MDSC, Myeloid-Derived Suppressor Cell; NK Cell, Natural Killer Cell; NKG2D, Natural Killer Group 2 Member D; PD-1, Programmed Cell Death Protein 1; PD-L1, Programmed Death-Ligand 1; SBRT, Stereotactic Body Radiotherapy; TAA, Tumor-Associated Antigen; TME, Tumor Microenvironment; Treg, Regulatory T Cell.

## 7 Genetic engineering of NK cells to enhance functionality

Genetic engineering techniques have played a pivotal role in enhancing NK cell efficacy. Strategies include constitutive expression of IL-2 or IL-15 to improve proliferation and persistence, promoting an activated receptor phenotype to augment cytotoxicity, and addressing the immunosuppressive TME. Retroviral transduction of NK-92 cells with IL-2 and membrane-bound IL-15 has demonstrated improved anticancer efficacy in both *in vivo* and *in vitro* models, along with enhanced persistence and proliferation (119).

Several approaches have been developed to increase NK cell numbers and function, such as using antibodies and cytokines to enhance NK activity (120), establishing homogeneous NK cell lines from healthy and cancer donors (121), adoptive transfer of allogeneic or autologous ex vivo expanded NK cells (122), and deriving NK cells from induced pluripotent stem cells (iPSCs) (123).

CAR expression using nonviral methods typically results in transient expression compared to stable gene expression via viral vectors. Recent innovations highlight the adaptability of lipid nanoparticles for delivering various RNA types, facilitating transient CAR generation with minimal toxicity. Preclinical findings suggest lipid nanoparticles as a promising alternative, warranting further evaluation in clinical trials (124).

Retroviral and lentiviral vectors integrate into the genome, offering long-term expression, whereas adenoviral vectors remain episomal and support only transient expression. Long-term CAR expression can also be achieved using transposon-based integration systems (42, 46).

Advancements in CAR NK cell research have highlighted genetic modifications that improve activation, persistence, and tumor killing efficacy. Strategies focus on costimulatory molecules, checkpoint modulation, and resistance to tumor-induced stressors, each contributing to the optimization of CAR NK cells for clinical use.

Key costimulatory molecules, such as DAP10, DAP12, CD80, and CD86, play crucial roles in enhancing NK cell functions. DAP10, an adaptor protein linked to NKG2D signaling, boosts cytotoxicity and metabolic fitness, leading to superior antitumor responses (125). Similarly, DAP12 strengthens activation and proliferation when incorporated into CAR engineering (125). CD80 and CD86 increase tumor cell susceptibility to NK-mediated lysis, serving as effective costimulatory signals (126). Nevertheless, challenges such as off-target effects and the complexity of targeting a diverse TME require careful optimization.

Checkpoint modulation remains a critical focus area. Immune checkpoints, such as PD-1, inhibit NK cell function, often through tumor-mediated acquisition, leading to diminished cytotoxicity (127). Disruption of checkpoints using CRISPR/Cas9, including NKG2A deletion, has shown promise in improving CAR NK cell activity (128). Integrating checkpoint inhibitors into CAR NK therapies, such as PD-L1-specific CARs, enhances cytotoxicity and cytokine production against both PD-L1-positive and negative tumors (129). Combining CAR NK therapy with dual-checkpoint inhibition, targeting PD-1 and CTLA-4, offers synergistic therapeutic benefits currently under investigation in clinical trials (130).

Recent efforts to overcome the hostile TME have included genetic modifications that bolster CAR NK cell survival and functionality. The constitutively active IL-7 receptor complex (C7R) improves survival and effector function by sustaining STAT5 activity independently of exogenous cytokines (131). Catalase engineering enables CAR NK cells to decompose hydrogen peroxide, mitigating oxidative stress and hypoxia in the TME, as demonstrated in triple-negative breast cancer models (132). Nonviral genome engineering techniques that silence inhibitory receptors further alleviate tumor-induced immunosuppression and restore cytotoxicity (133).

Despite significant progress, challenges persist in achieving consistent efficacy across various tumor types and managing patient-specific responses. Future research should prioritize refining genetic modifications, ensuring safety, and personalizing therapies. Overall, integrating costimulatory molecules, checkpoint modulation, and stress resistance mechanisms provides a strong foundation for advancing CAR NK cell-based cancer therapies.

### 7.1 Transduction of NK cells

Viral vectors play a critical role in gene editing techniques by enabling the repair of mutated or defective genes. Gene replacement strategies often depend on the *in vivo* administration of gene-bearing adeno-associated viruses. Compared to nonviral vectors, viral vectors are generally more efficient at inserting CAR-based genes into immune cells. Among viral vectors, lentiviral and retroviral vectors have proven to be the most effective for NK cell transduction due to their ability to integrate genetic material into the host genome, ensuring stable and long-term gene expression (134).

A recent clinical study focused on treating CD19+ non-Hodgkin’s lymphoma and chronic lymphocytic leukemia through the infusion of retrovirus-transduced anti-CD19 CAR cord blood NK cells. Approximately 73% of the patients responded positively to the treatment, with seven out of eight achieving complete remission. The study also observed prompt and consistent responses within a 30-day timeframe across all dosage levels. After one year of follow-up, enlarged C^4^ copies of the CAR NK vector per microgram of genomic DNA (63). These findings provide the first demonstration of long-term *in vivo* persistence of retrovirally transduced CAR NK cells.

Genetic engineering of NK cells to express specific chemokine receptors has significantly enhanced their migration toward tumor-associated chemokines, an essential mechanism for effective antitumor activity. This strategy exploits chemokine signaling pathways that regulate cell trafficking within the body, enabling NK cells to home more efficiently to tumor sites and lymphoid tissues where they exert cytotoxic effects. These modifications have improved the ability of NK cells to target and infiltrate tumors, overcoming one of the major limitations of natural NK cell trafficking in cancer therapy (135).

The development of scalable and efficient gene delivery methods has further facilitated these advancements. One such method, mRNA electroporation, has emerged as a robust technique for transiently expressing transgenes in NK cells (136). This technique introduces synthetic mRNA encoding the desired chemokine receptors into NK cells by temporarily permeabilizing the cell membrane through electroporation. Importantly, mRNA electroporation preserves NK cell viability and function while enabling rapid receptor expression. Studies using this method have consistently demonstrated improved NK cell homing capabilities, establishing it as a valuable tool for enhancing NK cell-based therapies (137).

In conclusion, advancements in gene therapy, including viral vector-based transduction and mRNA electroporation, have significantly enhanced the ability of NK cells to express tailored chemokine receptors and CAR constructs. **Table 3** summarizes the advantages and limitations of lentiviral, retroviral, CRISPR-Cas9, and nonviral methods for NK cell therapy based on factors such as efficiency, specificity, safety, cost, and scalability. These developments have improved tumor homing, sustained persistence, and effective antitumor activity in both preclinical and clinical settings.

**Table 3:** Comparison to understand the pros and cons of each gene editing approach for NK cell therapy.

Parameter	Lentiviral	Retroviral	CRISPR-Cas9	Nonviral Methods
Transduction Efficiency	High; efficient for dividing and non-dividing cells	Moderate; primarily effective in dividing cells	High; precise editing possible	Variable; depends on the method used
Duration of Gene Expression	Permanent, stable expression	Permanent, but insertional mutagenesis possible	Permanent for targeted genes	Transient or long-term, depending on delivery vector
Target Specificity	Moderate; integrates across a broad range of sites	Moderate; integrates across a broad range of sites	High; can be programmed for specific genes	Moderate; less specific compared to CRISPR
Ease of Use	Requires complex viral production processes	Similar complexity as lentiviral methods	High technical expertise needed	Easier for transient methods; may require optimization
Safety	Potential risk of insertional mutagenesis	Higher risk of insertional mutagenesis	Safer; off-target effects are a concern	Safer; lower risk of permanent genetic alterations
Cost	High	High	Moderate to high	Low to moderate
Scalability	Moderate; scalable but requires expertise	Moderate; scalable with established protocols	High; scalable with multiplexed editing	High; easier to scale in nonviral methods
Regulatory Challenges	High due to viral integration risks	High due to insertional mutagenesis risks	Moderate; concerns over editing accuracy	Low; fewer regulatory barriers
Suitability for NK Cells	High; widely used in NK cell engineering	Moderate; less efficient for NK cells	High; precise modifications in NK cells	Moderate; dependent on method and cell type

### 7.2 Transfection of NK cells

Lipofection, electroporation, and nucleofection are among the most commonly used nonviral methods for engineering NK cells (134). Transfection can be achieved using either naked plasmid DNA or mRNA, typically through electroporation (117). Lipofection, a strategy based on liposome encapsulation, is a well-established method for gene transfer. In this technique, liposomes containing the desired genes or proteins fuse with the target cell membrane and release their cargo into the cell (133).

Lipofection has been employed to transfer a plasmid encoding murine IL-2 into primary NK cells, using 1,2-dimyristyloxy-propyl-3-dimethyl-hydroxyethyl ammonium bromide/dioleoyl phosphatidylethanolamine (DMRIE/DOPE) as the reagent (133, 134). IL-2 enhances NK cell proliferation and cytotoxicity. Modified NK cells demonstrated a notable increase in granzyme A activity when used to treat melanoma xenografts. Furthermore, NK-92 cells transfected with stem cell factor cDNA via lipofection showed significantly increased proliferation and enhanced cytotoxicity compared to unmodified NK-92 cells, suggesting their potential in targeting various cancers (133).

Introduction of the microRNA miR-486-5p into primary NK cells via lipofection resulted in enhanced cytotoxicity, along with upregulated perforin and NKG2D expression. miR-486-5p targets and downregulates the insulin-like growth factor-1 receptor (IGF-1R), a key regulator of hepatocellular carcinoma progression. Lipofectamine 2000 has been utilized to transfect primary NK cells with an activating CAR targeting HER2 (138). According to Regis et al., miR-27a-5p negatively regulates CX3CR1, a chemokine receptor involved in guiding NK cells to peripheral organs and tumor sites. Using Lipofectamine 3000, a transfection efficiency of approximately 30% was achieved when primary NK cells were transfected with a miR-27a-5p inhibitor (133).

Electroporation relies on generating electrical pulses that create transient pores in the cell membrane, allowing charged molecules, DNA, RNA, and proteins to enter the cells (117, 134). When appropriate instruments and conditions are used, electroporation can achieve higher transfection efficiency compared to lipofection. However, the success of electroporation depends on the specific cell type. The viability of transfected cells is influenced by the nature and size of the delivered cargo. In NK cells, cytokines are necessary to facilitate transfection. Nevertheless, the low viability of many primary immune cells, including NK cells, limits transfection rates and efficiency (134).

Nucleofection techniques enable efficient gene transfer directly into the nucleus, independent of cell division (133). Electroporation and nucleofection hold potential for therapeutic applications and are often considered superior to lipofection (134). For electroporation, cells need to be in the exponential growth phase to allow optimal nuclear access (139). Several studies have demonstrated the potential of (140, 141), offering transient gene expression and reduced risk of genomic integration.

Nanoparticles have emerged as an alternative nonviral transfection strategy to overcome the limitations of lipofection and electroporation. Various types of nanoparticles, including polymer-based, lipid-based, and inorganic nanoparticles, have been developed to protect genetic material and enhance cellular uptake (30, 142). Iron oxide core magnetic nanoparticles (MNPs) have been used to transfect NK-92 cells, resulting in MNP-modified NK cells with improved tumor targeting capabilities (143). These nanoparticles can attach to the cell surface, enhancing transfection efficiency in primary NK cells. However, the toxicity associated with high doses of nanoparticles remains a major limitation.

Transposons offer a stable, nonviral method for gene delivery (144). As repetitive DNA sequences capable of mobilization within the genome, transposons provide an alternative to viral vectors for engineering human cells. They are currently being used in clinical trials for stable gene transfer in transposon-engineered T cells and iPSCs (145–147).

The system produces a complex through the pairing of tracer RNA with palindromic sequences and the assembly of crRNA, tracer RNA, and Cas9 protein. RNase III cleaves the complex, enabling crRNA to guide Cas9 for site-specific double-strand breaks (134). CRISPR-Cas9 precisely edits genes at specific loci with the help of guide RNAs that direct Cas9 to the target site. The system can simultaneously edit multiple genes using multiple single-guide RNAs (148).

Gene silencing through shRNA and siRNA is another well-established approach, used to downregulate target gene expression and enhance NK cell cytotoxicity in therapeutic applications. Lentiviral transduction and electroporation have been utilized for this purpose. siRNA strategies have improved the anticancer potential of NK cells by overcoming NK cell exhaustion and suppressing the inhibitory receptor NKG2A in cancer immunotherapy.

## 8 Improving NK cell homing and migration to tumor site

The migration and localization of NK cells are governed by cell-extrinsic elements (including signals from sphingosine-1-phosphate, cytokines, chemokines, selectins, integrins, and associated receptors), cell-intrinsic elements, and the microenvironment (149).

Integrins are transmembrane receptors composed of 18 α subunits and eight β subunits that form heterodimers. These heterodimers bind various extracellular ligands, including selectins, cell adhesion molecules, and more than 20 ECM components (150). NK cells express diverse integrins critical for motility and tissue residency and also serve as phenotypic markers distinguishing resident from non-resident subsets (151). The structural and functional adaptability of integrins allows cells to regulate migration speed and direction (152).

β1 integrins, widely expressed beyond leukocytes, contribute to target cell cytotoxicity and tissue infiltration. NK cells frequently express markers such as α4/β1, αL/β2, α5/β1, α2/β1, αM/β2, and α1/β1, enabling interactions with molecules including MAdCAM-1, vitronectin, fibronectin, connective tissue proteins, basement membrane components, epithelial cadherin, and vascular adhesion molecule-1 (153). β2 integrins support adhesion and immunological synapse formation during tumor cell lysis and facilitate NK cell trafficking between tissues and blood (43). Specific β2 integrins—αDβ2, αX/β2, αM/β2, and αL/β2—interact with CAM family proteins like MAdCAM, ICAM, and VCAM in lymphoid and non-lymphoid environments (151).

Within the bone marrow, NK cells reside in both sinusoids and parenchyma. During egression, they move from the parenchyma into sinusoids and subsequently into circulation. Immature subsets, including NKPs and iNKs, tend to remain in the parenchyma, characterized by high CXCR4 expression. As NK cells mature, CXCR4 levels decline, enabling migration to peripheral tissues. Conversely, increased CXCR4 levels promote retention within the bone marrow (43). After exiting bone marrow sinusoids and circulating via the bloodstream, NK cells migrate to peripheral and secondary lymphoid tissues, guided by SIP5 and CX3CR1 (154).

NK cells more readily infiltrate hematopoietic malignancies compared to solid tumors. Infiltration of solid tumors presents additional challenges which requires extravasation followed by navigation through the ECM and tumor stroma (155). NK cells employ several mechanisms to traverse this barrier, including degradation of ECM components by enzymes such as serine dipeptidyl peptidase IV, matrix urokinase plasminogen activator, and metalloproteinases. Chemokines, selectins, and integrins support this process by directing NK cells toward tumor sites (156). Upon receptor-ligand interactions—such as CCL5-CCR5, CCL27-CCR10, and CX3CL1-CX3CR1—NK cells engage other immune cells and initiate anticancer responses via ADCC, degranulation, and apoptosis through FASL or TRAIL pathways (157). These chemokine axes have been implicated in NK cell recruitment to the TME in both humans and mice (157).

The cytotoxic activity of NK cells can be impaired by direct cell–cell contact or soluble inhibitory factors within the TME. Cancer and stromal cells release factors that reprogram NK cells, promoting tumor angiogenesis and facilitating immune evasion. This transformation shifts the immune balance toward a pro-tumor state (82). Once inside the TME, NK cells exhibit altered phenotypes and metabolic profiles, including increased expression of exhaustion markers such as CD96, PD-1, TIGIT, and Tim3, along with decreased expression of activating receptors like NKp80, CD16, DNAM1, and NKp30 (158). Soluble factors such as IDO, IL-10, PGE2, and TGF-β, secreted by carcinoma-associated fibroblasts, tumor cells, Tregs, and others, further suppress NK cell effector functions (148). Additionally, receptor-ligand interactions—such as 2B4-CD48 and NKG2A-HLAE—between NK cells and various immunosuppressive cells, including tumor cells, Tregs, and myeloid-derived suppressor cells, contribute to functional inhibition (158).

## 9 Gene editing techniques to improve CAR NK cell therapy

Gene editing technologies, including CRISPR/Cas9, TALENs, and zinc finger nucleases, have transformed cancer immunotherapy by allowing precise genetic alterations that enhance immune cell function. Although T cell engineering has shown substantial clinical success, modifying NK cells genetically has faced obstacles due to poor transfection efficiency, a tendency toward apoptosis, and challenges in maintaining stable gene expression.

Recent developments in gene editing have enhanced NK cell cytotoxicity, durability, and tumor targeting capacity. CRISPR/Cas9 has enabled the knockout of inhibitory receptors such as cytokine-inducible SH2-containing protein (CISH) to increase NK cell responsiveness (159). It has also supported the insertion of CAR constructs (160), cytokine-related genes like IL-15 (161), and chemokine receptors such as CXCR4 (162) to improve NK cell homing to tumors.

Gene editing can also reinforce resistance to the suppressive tumor microenvironment by disrupting checkpoint regulators like PD-1, TIGIT, and TGF-β receptors, limiting tumor-mediated inhibition of NK cells. Transposon-based platforms, including Sleeping Beauty and PiggyBac, offer nonviral strategies for stable gene integration, reducing the risks tied to viral vectors (163). These approaches aim to produce CAR NK cell therapies with greater stability, specificity, and antitumor performance across both hematologic and solid malignancies.

## 10 Preclinical studies on CAR NK cell therapy

Preclinical strategies to enhance NK cell-mediated tumor elimination include the incorporation of CARs to redirect cytotoxicity, suppression of inhibitory receptors such as NKG2A to improve tumor specificity, and stimulation of NK cell persistence *in vivo* through autocrine cytokine signaling with IL-2 and IL-15 (22). The introduction of CAR constructs into NK cells has received considerable attention, prompting extensive investigation over the past decade. These efforts have included *in vitro* experiments and *in vivo* studies using murine xenograft models. Research has focused on various NK cell sources, methods for cell expansion, genetic modification, and different plasmid constructs and vector systems (26).

Allogeneic haploidentical NK cells offer advantages over CAR T cells due to their distinct biological attributes. A key advantage of CAR NK cells is their favorable safety profile. CAR T therapies are commonly associated with toxicities such as neurotoxicity and CRS, which can present as hypoxia, hypotension, sinus tachycardia, hyperpyrexia, cardiac dysfunction, and multiorgan failure (72, 79). These toxicities are mainly driven by pro-inflammatory cytokines, including IL-1, IL-6, and TNFα, whereas CAR NK cells primarily release cytokines such as GM-CSF and IFN-γ (41). CAR T cells, whether autologous or allogeneic, carry a risk of GvHD due to HLA incompatibility. In contrast, NK cells can trigger early graft-versus-leukemia effects and potentially suppress GvHD by targeting recipient cytotoxic T lymphocytes and APCs (72).

CAR NK cells demonstrate enhanced antitumor activity compared to CAR T cells by leveraging both innate and engineered cytotoxic mechanisms. CAR integration can augment NK cell specificity and potency against tumor antigens. Unlike CAR T cells, CAR NK cells retain their intrinsic ability to kill even when tumor antigen expression is reduced (28). CAR NK cells are easier to produce than CAR T cells due to lower GvHD risk and broader donor compatibility, including HLA-matched or mismatched sources. This enables standardized, off-the-shelf therapies. Ongoing clinical trials are assessing their safety and efficacy in both hematologic and solid tumors, using diverse NK sources (e.g., cord blood, iPSCs) and targeting antigens like CD19, BCMA, and PD-L1. Many protocols incorporate lymphodepletion to boost cell persistence. These foundational studies set the stage for the clinical advancements discussed later.

## 11 Clinical studies on CAR NK cell therapy

The ongoing trials (**Table 4**) differ in design, phase, and recruitment status, reflecting the early developmental stage of CAR NK therapies but also highlighting growing interest in their clinical application. For example, FT596, an off-the-shelf, IL-15–enhanced CAR NK cell targeting CD19, has shown good tolerability and early clinical responses in relapsed B-cell lymphoma (NCT04245722). PD-1–deleted CAR NK cells targeting mesothelin are under evaluation in solid tumors like ovarian and pancreatic cancers, with early results showing enhanced persistence and no severe CRS (NCT05732948). CYNK-001, derived from placental NK cells, is being tested in AML with manageable side effects and promising off-the-shelf applicability (NCT04310592). In glioblastoma, CAR NK92 cells targeting HER2 were found to be safe and associated with disease stabilization (NCT03383978). Finally, CD7-targeted CAR NK cells are being explored in T-cell malignancies with encouraging results including rapid expansion and cytotoxicity without NK fratricide (NCT05020678). Compared to CAR T cells, CAR NK cells have shown a more favorable safety profile in these trials, with minimal reports of CRS or neurotoxicity, supporting their potential as a safer and scalable alternative for cancer immunotherapy.

**Table 4:** Current progress of ongoing clinical trials of CAR NK cells.

Sr.No.	Intervention	Cancer type	NK source	Start year	Location	Target	Status	Trial phase	Clinical phase
1	PD-L1 t-haNK	Metastatic Solid Cancers	Not specified	18-Jul-19	United States	PD-L1	Active, not recruiting	I	NCT04050709
2	Cells (ROBO1 CAR NK cells)/PD-L1 t-haNK	Locally Advanced or Metastatic Pancreatic Cancer	Not specified	21-Jul-20	United States	PD-L1-expressing cells	Recruiting	II	NCT04390399
3	BiCAR-NK/T	Malignant Tumors	Not specified	May-19	China	ROBO1	Recruiting	I/II	NCT03931720
4	NKX019	Lymphoma, NonHodgkin; B cell Acute Lymphoblastic Leukemia; Large B cell Lymphoma	Allogenic source	20-Aug-21	United States, Australia	CD19	Recruiting	I	NCT05020678
5	Drug: Fludarabine + Cyclophosphamide + CAR NK-CD19 Cells	Acute Lymphocytic Leukemia; Chronic Lymphocytic Leukemia; Nonhodgkin’s Lymphoma	Cord blood-derived NK cells	10-Apr-21	China	CD19	Recruiting	I	NCT04796675
6	Anti-BCMA CAR NK Cells	Multiple Myeloma, Refractory	Umbilical & Cord Blood-derived NK cells	01-Oct-21	China	BCMA	Not Yet Recruiting	Early I	NCT05008536
7	NKX101-CAR NK Cell Therapy	Relapsed/Refractory AML; AML, Adult MDS; Refractory Myelodysplastic Syndromes	off-the-shelf donor-derived NKX101	21-Sep-20	United States	NKG2D Ligands	Recruiting	I	NCT04623944
8	Anti-CD33 CAR NK cells, Drug Fludarabine, Drug Cytoxan	Leukemia, Myeloid, Acute	Not specified	23-Dec-21	China	CD33	Not Yet Recruiting	I	NCT05008575
9	Drug: FT596 Experimental Interventional Therapy	B cell lymphoma, CLL	iPSCs (FT596)	19-Mar-20	United States	CD19	Recruiting	I	NCT04245722
10	CD19 t-haNK	Diffuse large B cell lymphoma	NK92	16-Sep-19	United States	CD19	Not Yet Recruiting	I	NCT04052061
11	Anti-CD19 CAR NK cells	B Cell Nonhodgkin Lymphoma	Not specified	01-May-21	China	CD19	Recruiting	I	NCT04887012
12	Anti-CD19 CAR NK cells	B cell NHL	HLA haploidentical NK cells	17-Dec-20	China	CD19	Not Yet Recruiting	Early I	NCT04639739
13	Drug: FT596 Drug: Rituximab	B cell lymphoma	iPSCs (FT596)	22-Sep-20	United States	CD19	Recruiting	I	NCT04555811
14	Anti-CD19 iCAR NK Cells	B cell NHL	iPSCs	01-Feb-19	N/A	CD19	Not Yet Recruiting	Early I	NCT03824951

PD-L1, Programmed Death-Ligand 1; AML, Acute Myeloid Leukemia; BCMA, B Cell Maturation Antigen; BiCAR, Bispecific Chimeric Antigen Receptor; CAR, Chimeric Antigen Receptor; CD19, Cluster of Differentiation 19; CD33, Cluster of Differentiation 33; CLL, Chronic Lymphocytic Leukemia; FT596, Fate Therapeutics Cell Product 596; HLA, Human Leukocyte Antigen; iCAR, Inhibitory Chimeric Antigen Receptor; iPSCs, Induced Pluripotent Stem Cells; MDS, Myelodysplastic Syndromes; NHL, Non-Hodgkin Lymphoma; NK, Natural Killer; NKG2D, Natural Killer Group 2 Member D; NKX019, Natural Killer Cell Experimental Therapy 019; ROBO1, Roundabout Guidance Receptor 1; t-haNK, Targeted High Affinity Natural Killer cells.

## 12 Future directions and challenges

**Figure 4:**
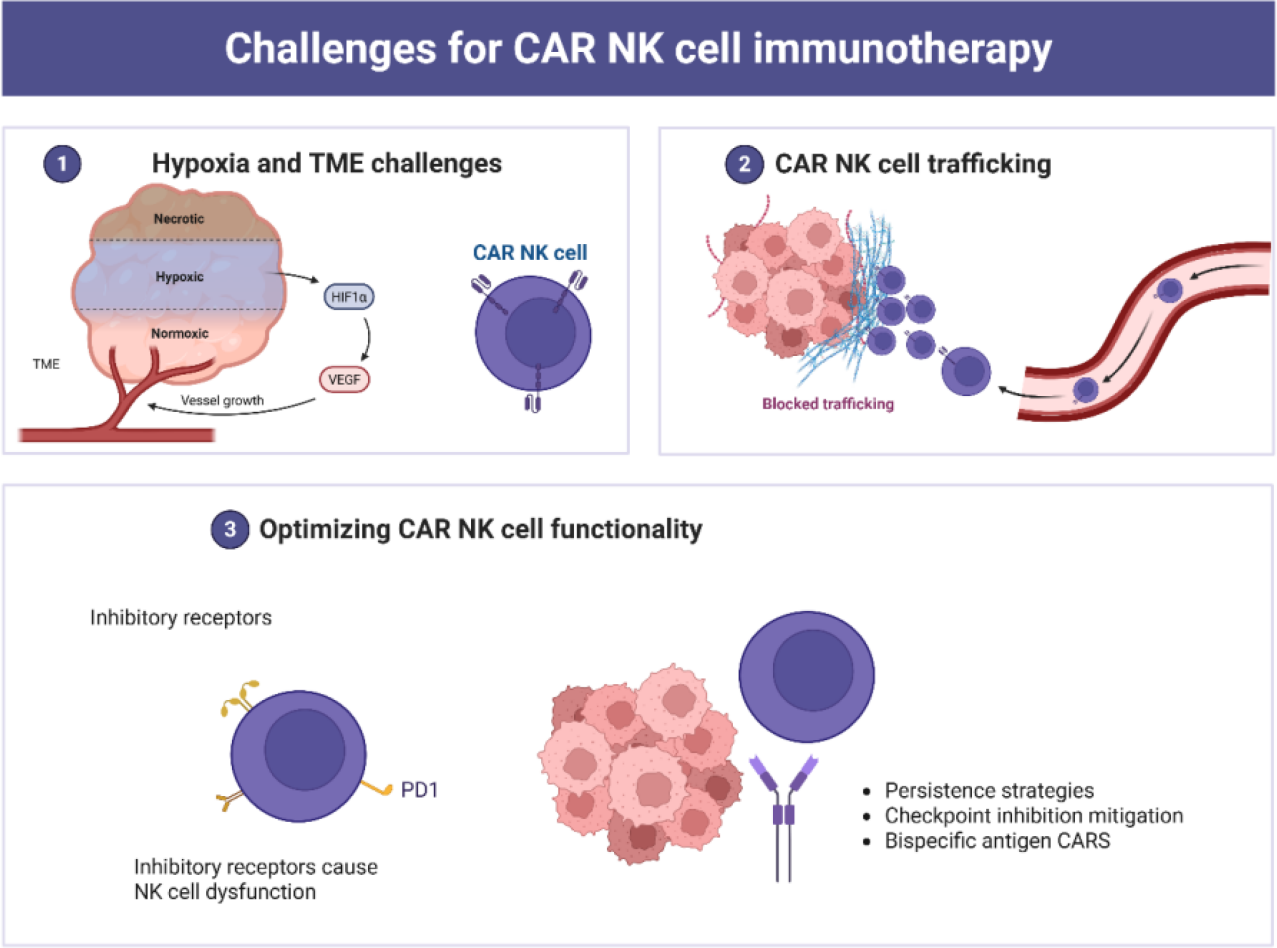
Multifaceted challenges associated with CAR NK cell immunotherapy. (1) The TME poses a significant barrier, with hypoxic, necrotic and normoxic regions, promoting immune evasion and angiogenesis and reducing CAR NK cell adaptability. (2) Trafficking and infiltration barriers limit CAR NK cell access to solid tumors due to the dense TME; and (3) Functional optimization strategies, including enhanced persistence, checkpoint inhibition mitigation, and bispecific antigen CARs, aim to improve therapeutic efficacy and adaptability in complex TMEs.

CAR NK cells are considered promising agents in tumor immunotherapy. However, several limitations hinder their effectiveness, including antigen loss, inefficient trafficking to tumor sites, tumor heterogeneity, low persistence, and the immunosuppressive nature of the TME (**Figure 4**). NK cells display heightened sensitivity to freezing and thawing compared to T lymphocytes and other human cell types (26, 72). This vulnerability, along with limited expansion and persistence, poses logistical challenges for the scalable and timely distribution of NK cells. Consequently, the clinical use of engineered NK cell products remains largely confined to adoptive immunotherapy. Developing optimized cryopreservation protocols is essential for improving freezing and banking strategies.

Various strategies have been implemented to overcome trafficking barriers in solid tumors. These include intraperitoneal and local administration, as well as ultrasound-guided delivery systems (164). Nevertheless, regional delivery may result in suboptimal therapeutic dosing (28). Inhibitory receptors also contribute to NK cell dysfunction. These include cytokine checkpoints (*e.g.*, CISH), C-type lectin receptors (*e.g.*, NKG2A), and immune checkpoints (*e.g.*, CTLA-1, PD-1, TIGIT) (25). Additionally, immunosuppressive factors present in the TME, such as indoleamine 2,3-dioxygenase, adenosine, IL-10, arginase, and TGF-β, impair CAR NK cell functionality (25, 28, 72).

TGF-β inhibition strategies have demonstrated potential in preserving NK cell cytotoxicity and receptor expression. Co-administering NK cells with TGF-β kinase inhibitors helps maintain CD16 and NKG2D expression (28). Similarly, agents such as alisertib (a TGF-βRI inhibitor) and fresolimumab (a neutralizing antibody against TGF-β) have shown promise in solid tumors (165).

The TME is characterized by nutrient deficiency, hypoxia, and acidosis, which suppress immune responses (166). Hypoxia disrupts metabolic balance and upregulates tumor-promoting factors and angiogenesis. It also reduces the expression of activating receptors on NK cells, including NKp44, NKp30, NKG2D, and NKp46 (28). Arginase, activated under hypoxic conditions through CD73, further diminishes NK cell activity. Augmented homing of NKG2D-CAR NK cells has enhanced antitumor efficacy in lung cancer models (28). Hybrid CARs incorporating TGF-β receptor domains have similarly improved NK-92 cell activity (167).

Immune checkpoints naturally regulate NK cell responses to prevent overactivation and autoimmunity. Tumor cells exploit this mechanism by upregulating checkpoint ligands, thereby inhibiting NK cell function. Genetic deletion or blockade of these checkpoints enhances CAR NK cell activity and accelerates tumor clearance. For instance, TIGIT suppresses NK cytotoxicity by countering CD226 (28). Knocking down SMAD3, a TGF-β downstream effector, has also improved NK cytotoxic potential (18). Dominant-negative TGF-β receptors allow UCB-NK cells to maintain IFN-γ secretion and glioblastoma cell killing. Despite tumor hypoxia, entinostat increases NKG2D in NK cells and MICA in tumor cells, improving recognition and killing.

Engineering CAR NK cells to enhance functionality and therapeutic potential is vital. Cytokines such as IL-2, IL-12, and IL-15 support NK cell expansion, persistence, and cytotoxicity in both innate and adoptive contexts. IL-2 supplementation can restore the functionality of cryopreserved NK cells. Genetic strategies that involve deleting inhibitory genes and incorporating cytokine-encoding constructs offer promising avenues. The integration of suicide genes into CAR NK cells provides an important safety measure to counteract excessive cytokine release. The activation of the iC9 suicide gene has enabled the effective elimination of C9/CAR.19/IL-15 CB-NK cells in both preclinical and clinical settings (63).

In lymphoma models, CD19 UCB-derived CAR NK cells engineered to secrete IL-15 and lacking CISH have shown improved metabolic fitness and antitumor activity (26). Most TAAs are not exclusive to tumor cells and are also expressed on healthy tissues, complicating target specificity and increasing the risk of ‘on-target, off-tumor’ toxicity. Therefore, CARs must be designed to recognize highly expressed, tumor-specific antigens (52). Intra-tumoral heterogeneity and clonal evolution further decrease uniform TAA expression, necessitating the development of bispecific CARs that can recognize multiple targets simultaneously.

Genetic modification of NK cells to express CARs and auxiliary genes has given rise to multifunctional constructs such as “armored” CAR NK cells or “NK cell pharmacies” (168). These constructs have demonstrated susceptibility to pharmacological elimination via iC9 suicide gene activation in both preclinical and clinical studies (63). NK cell potency can be enhanced by monoclonal antibodies, ICB, and cytokines in adoptive immunotherapy protocols (72). Radiotherapy has also been shown to upregulate NKG2D ligands through DNA methylation, further enhancing CAR NK cell cytotoxicity. Pre-infusion lymphodepletion through chemotherapy or radiation improves the effector-to-target ratio and reduces tumor burden, thereby enhancing the overall effectiveness of CAR NK therapy.

Current expansion techniques remain limited, as only a few methods yield sufficient NK cell numbers for clinical use (103). Feeder cell-based expansion, often involving cancer cell lines, carries a risk of contamination. Additionally, NK cells exhibit lower transduction and transfection efficiencies compared to other immune cells, complicating consistent genetic engineering outcomes. Despite these limitations, NK cells possess innate cytotoxic abilities independent of CAR signaling. Enhancing homing and targeting efficiency can be achieved by modifying non-signaling CAR regions to include adhesion molecules and chemokine receptors, rather than relying solely on antigen recognition. This approach may be particularly effective in hematologic malignancies such as lymphoma, where the tumor site is more accessible.

The “missing-self” recognition mechanism offers a safety advantage, as NK cells can discriminate between healthy and malignant cells, minimizing off-target effects. Selecting tumor-specific TAAs is critical to avoid cytotoxicity toward normal tissues. T cell and myeloid malignancies often share antigens with healthy cells, increasing toxicity risk (169). NKG2D has emerged as a promising target, and its safety has been demonstrated in the treatment of multiple myeloma and myelodysplastic Syndromes/Acute Myeloid Leukemia using NKG2D-based CAR NK cells.

## 13 Conclusion

Despite promising outcomes in both preclinical and clinical studies, NK cell-based tumor immunotherapy continues to encounter several critical challenges. These include immune evasion by tumors, a suppressive tumor microenvironment, tumor heterogeneity, reduced cytotoxic function, low expression of TAAs, and poor trafficking to tumor sites, all of which compromise *in vivo* persistence. The short lifespan and limited curative efficacy of adoptively transferred NK cells remain major barriers to effective cancer treatment. Although CAR NK cell therapy has shown encouraging results, it is still in the early stages of development compared to CAR T cell therapy. Optimizing gene constructs, refining delivery systems, and improving methods for generating engineered NK cells are areas under active investigation. Addressing these limitations is essential for advancing NK cell-based cancer immunotherapy toward broader clinical application.

## References

[1] Siegel RL Kratzer TB Giaquinto AN Sung H Jemal A. Cancer statistic. CA: A Cancer J Clinicians. (2025) 75:10–45. doi: 10.3322/caac.21871 PMC11745215 39817679

[2] Afzal A Abbasi MH Ahmad S Sheikh N Khawar MB. Current trends in messenger RNA technology for cancer therapeutics. Biomaterials Res. (2025) 29:0178. doi: 10.34133/bmr.0178 PMC11978394 40207255

[3] Su L Chen G Liu Z Min Y Wang AZ. Delivery strategies to overcome tumor immunotherapy resistance. Systemic Drug Delivery Strategies. Amsterdam, Netherlands: Elsevier (2022) p. 529–47.

[4] Siegler EL Zhu Y Wang P Yang L. Off-the-shelf CAR-NK cells for cancer immunotherapy. Cell Stem Cell. (2018) 23:160–1. doi: 10.1016/j.stem.2018.07.007 30075127

[5] Esfahani K Roudaia L Buhlaiga N Del Rincon S Papneja N Miller W. A review of cancer immunotherapy: from the past, to the present, to the future. Curr Oncol. (2020) 27:87–97. doi: 10.3747/co.27.5223 PMC7194005 32368178

[6] Robert C. A decade of immune-checkpoint inhibitors in cancer therapy. Nat Commun. (2020) 11:3801. doi: 10.1038/s41467-020-17670-y 32732879 PMC7393098

[7] Saxena M van der Burg SH Melief CJM Bhardwaj N. Therapeutic cancer vaccines. Nat Rev Cancer. (2021) 21:360–78. doi: 10.1038/s41568-021-00346-0 33907315

[8] Morotti M Albukhari A Alsaadi A Artibani M Brenton JD Curbishley SM. Promises and challenges of adoptive T-cell therapies for solid tumours. Br J Cancer. (2021) 124:1759–76. doi: 10.1038/s41416-021-01353-6 PMC8144577 33782566

[9] Chyuan I-T Chu C-L Hsu P-N. Targeting the tumor microenvironment for improving therapeutic effectiveness in cancer immunotherapy: focusing on immune checkpoint inhibitors and combination therapies. Cancers. (2021) 13:1188. doi: 10.3390/cancers13061188 33801815 PMC7998672

[10] Stein-Merlob AF Rothberg MV Holman P Yang EH. Immunotherapy-associated cardiotoxicity of immune checkpoint inhibitors and chimeric antigen receptor T cell therapy: diagnostic and management challenges and strategies. Curr Cardiol Rep. (2021) 23:1–11. doi: 10.1007/s11886-021-01440-3 PMC7821837 33483873

[11] Labanieh L Mackall CL. CAR immune cells: design principles, resistance and the next generation. Nature. (2023) 614:635–48. doi: 10.1038/s41586-023-05707-3 36813894

[12] Khawar MB Afzal A Abbasi MH Sheikh N Sun H. Nano-immunoengineering of CAR-T cell therapy against tumor microenvironment: The way forward in combating cancer. OpenNano. (2023) 10:100124. doi: 10.1016/j.onano.2023.100124

[13] Shalhout SZ Miller DM Emerick KS Kaufman HL. Therapy with oncolytic viruses: progress and challenges. Nat Rev Clin Oncol. (2023) 20:160–77. doi: 10.1038/s41571-022-00719-w 36631681

[14] Yang K Halima A Chan TA. Antigen presentation in cancer—mechanisms and clinical implications for immunotherapy. Nat Rev Clin Oncol. (2023) 20:604–23. doi: 10.1038/s41571-023-00789-4 37328642

[15] Klobuch S Seijkens TTP Schumacher TN Haanen JBAG. Tumour-infiltrating lymphocyte therapy for patients with advanced-stage melanoma. Nat Rev Clin Oncol. (2024) 21:173–84. doi: 10.1038/s41571-023-00848-w 38191921

[16] Blass E Ott PA. Advances in the development of personalized neoantigen-based therapeutic cancer vaccines. Nat Rev Clin Oncol. (2021) 18:215–29. doi: 10.1038/s41571-020-00460-2 PMC7816749 33473220

[17] De Bousser E Callewaert N Festjens N. T cell engaging immunotherapies, highlighting chimeric antigen receptor (CAR) T cell therapy. Cancers. (2021) 13:6067. doi: 10.3390/cancers13236067 34885176 PMC8657024

[18] Wang Q-M Tang PM-K Lian G-Y Li C Li J Huang X-R. Enhanced cancer immunotherapy with Smad3-silenced NK-92 cells. Cancer Immunol Res. (2018) 6:965–77. doi: 10.1158/2326-6066.CIR-17-0491 29915022

[19] Senior K. Making CAR T-cell therapies more affordable. Lancet. (2025) 405:187–8. doi: 10.1016/S0140-6736(24)02719-3 39681129

[20] Yang Z Ha B Wu Q Ren F Yin Z Zhang H. Expanding the horizon of CAR T cell therapy: from cancer treatment to autoimmune diseases and beyond. Front Immunol Volume. (2025) 16:2025. doi: 10.3389/fimmu.2025.1544532 PMC11880241 40046061

[21] Afzal A Khawar MB. CAR T therapies: game changer or culprit in cancer treatment? Albus Scientia. (2024) 2024:1–3. doi: 10.56512/AS.2024.1.e240305

[22] Laskowski TJ Biederstädt A Rezvani K. Natural killer cells in antitumour adoptive cell immunotherapy. Nat Rev Cancer. (2022) 22:557–75. doi: 10.1038/s41568-022-00491-0 PMC9309992 35879429

[23] Lv D Khawar MB Liang Z Gao Y Sun H. Neoantigens and NK cells:”Trick or treat” the cancers? Front Immunol. (2022) 13:931862. doi: 10.3389/fimmu.2022.931862 35874694 PMC9302773

[24] Taefehshokr S Parhizkar A Hayati S Mousapour M Mahmoudpour A Eleid L. Cancer immunotherapy: Challenges and limitations. Pathology-Research Pract. (2022) 229:153723. doi: 10.1016/j.prp.2021.153723 34952426

[25] Maskalenko NA Zhigarev D Campbell KS. Harnessing natural killer cells for cancer immunotherapy: dispatching the first responders. Nat Rev Drug Discov. (2022) 21:559–77. doi: 10.1038/s41573-022-00413-7 PMC10019065 35314852

[26] Daher M Rezvani K. Outlook for new CAR-based therapies with a focus on CAR NK cells: what lies beyond CAR-engineered T cells in the race against cancer. Cancer Discov. (2021) 11:45–58. doi: 10.1158/2159-8290.CD-20-0556 33277313 PMC8137521

[27] Pahl JHW Koch J Götz J-J Arnold A Reusch U Gantke T. CD16A activation of NK cells promotes NK cell proliferation and memory-like cytotoxicity against cancer cells. Cancer Immunol Res. (2018) 6:517–27. doi: 10.1158/2326-6066.CIR-17-0550 29514797

[28] Khawar MB Sun H. CAR-NK cells: from natural basis to design for kill. Front Immunol. (2021) 12. doi: 10.3389/fimmu.2021.707542 PMC8712563 34970253

[29] Khawar MB Gao G Rafiq M Shehzadi A Afzal A Abbasi MH. Breaking down barriers: The potential of smarter CAR-engineered NK cells against solid tumors. J Cell Biochem. (2023) 124:1082–104. doi: 10.1002/jcb.v124.8 37566723

[30] Khawar MB Afzal A Dong S Si Y Sun H. Engineering and targeting potential of CAR NK cells in colorectal cancer. Chin Med J. (2024) 10:1097. doi: 10.1097/CM9.0000000000003346 PMC12233927 39497428

[31] Yang C Wang Y Liu T Jiang F Wang Q Wang Q. Abstract 4078: Optimized chimeric antigen receptors (CARs) for CAR-NK cell therapies. Cancer Res. (2023) 83:4078–8. doi: 10.1158/1538-7445.AM2023-4078

[32] Huang R Wen Q Zhang X. CAR-NK cell therapy for hematological Malignancies: recent updates from ASH 2022. J Hematol Oncol. (2023) 16:35. doi: 10.1186/s13045-023-01435-3 37029381 PMC10082521

[33] Lin X Sun Y Dong X Liu Z Sugimura R Xie G. IPSC-derived CAR-NK cells for cancer immunotherapy. Biomed Pharmacother. (2023) 165:115123. doi: 10.1016/j.biopha.2023.115123 37406511

[34] Khawar MB Ge F Afzal A Sun H. From barriers to novel strategies: smarter CAR T therapy hits hard to tumors. Front Immunol. (2023) 14:1203230. doi: 10.3389/fimmu.2023.1203230 37520522 PMC10375020

[35] Xiong Q Zhu J Zhang Y Deng H. CAR-NK cell therapy for glioblastoma: what to do next? Front Oncol. (2023) 13. doi: 10.3389/fonc.2023.1192128 PMC10315652 37404752

[36] Kiessling R Klein E Pross H Wigzell H. “Natural” killer cells in the mouse. II. Cytotoxic cells with specificity for mouse Moloney leukemia cells. Characteristics of the killer cell. Eur J Immunol. (1975) 5:117–21. doi: 10.1002/eji.1830050209 1086218

[37] Kiessling R Klein E Wigzell H. “Natural” killer cells in the mouse. I. Cytotoxic cells with specificity for mouse Moloney leukemia cells. Specificity and distribution according to genotype. Eur J Immunol. (1975) 5:112–7. doi: 10.1002/eji.1830050208 1234049

[38] Jondal M Pross H. Surface markers on human B and T lymphocytes. VI. Cytotoxicity against cell lines as a functional marker for lymphocyte subpopulations. Int J Cancer. (1975) 15:596–605. doi: 10.1002/ijc.2910150409 806545

[39] Rosenberg EB McCoy JL Green SS Donnelly FC Siwarski DF Levine PH. Destruction of human lymphoid tissue-culture cell lines by human peripheral lymphocytes in 51Cr-release cellular cytotoxicity assays. J Natl Cancer Institute. (1974) 52:345–52. doi: 10.1093/jnci/52.2.345 4131425

[40] Whiteside TL. Chapter 10 - Natural killer (NK) cells. In: Fink G, editor. Stress: immunology and inflammation. Cambridge, Massachusetts: Academic Press (2024). p. 83–90.

[41] Xie G Dong H Liang Y Ham JD Rizwan R Chen J. CAR-NK cells: A promising cellular immunotherapy for cancer. EBioMedicine. (2020) 59:102975. doi: 10.1016/j.ebiom.2020.102975 32853984 PMC7452675

[42] Schmidt P Raftery MJ Pecher G. Engineering NK cells for CAR therapy—recent advances in gene transfer methodology. Front Immunol. (2021) 11:611163. doi: 10.3389/fimmu.2020.611163 33488617 PMC7817882

[43] Ran GH Lin YQ Tian L Zhang T Yan DM Yu JH. Natural killer cell homing and trafficking in tissues and tumors: from biology to application. Signal transduction targeted Ther. (2022) 7:205. doi: 10.1038/s41392-022-01058-z PMC9243142 35768424

[44] Marofi F Al-Awad AS Sulaiman Rahman H Markov A Abdelbasset WK Ivanovna Enina Y. CAR-NK cell: a new paradigm in tumor immunotherapy. Front Oncol. (2021) 11:673276. doi: 10.3389/fonc.2021.673276 34178661 PMC8223062

[45] Myers JA Miller JS. Exploring the NK cell platform for cancer immunotherapy. Nat Rev Clin Oncol. (2021) 18:85–100. doi: 10.1038/s41571-020-0426-7 32934330 PMC8316981

[46] Shin MH Kim J Lim SA Kim J Kim S-J Lee K-M. NK cell-based immunotherapies in cancer. Immune network. (2020) 20:e14. doi: 10.4110/in.2020.20.e14 32395366 PMC7192832

[47] Kumar AR Devan AR Nair B Vinod BS Nath LR. Harnessing the immune system against cancer: current immunotherapy approaches and therapeutic targets. Mol Biol Rep. (2021) 48:1–21. doi: 10.1007/s11033-021-06752-9 PMC8605995 34671902

[48] Zhuang X Long EO. NK cells equipped with a chimeric antigen receptor that overcomes inhibition by HLA class I for adoptive transfer of CAR-NK cells. Front Immunol. (2022) 13:840844. doi: 10.3389/fimmu.2022.840844 35585985 PMC9108249

[49] Vitale M Cantoni C Della Chiesa M Ferlazzo G Carlomagno S Pende D. An historical overview: the discovery of how NK cells can kill enemies, recruit defense troops, and more. Front Immunol. (2019) 10:1415. doi: 10.3389/fimmu.2019.01415 31316503 PMC6611392

[50] Cao Y Wang X Jin T Tian Y Dai C Widarma C. Immune checkpoint molecules in natural killer cells as potential targets for cancer immunotherapy. Signal transduction targeted Ther. (2020) 5:250. doi: 10.1038/s41392-020-00348-8 PMC7596531 33122640

[51] Guerra L Bonetti L Brenner D. Metabolic modulation of immunity: a new concept in cancer immunotherapy. Cell Rep. (2020) 32(1):107848. doi: 10.1016/j.celrep.2020.107848 32640218

[52] Wang J Liu X Jin T Cao Y Tian Y Xu F. NK cell immunometabolism as target for liver cancer therapy. Int Immunopharmacol. (2022) 112:109193. doi: 10.1016/j.intimp.2022.109193 36087507

[53] Terrén I Orrantia A Mikelez-Alonso I Vitallé J Zenarruzabeitia O Borrego F. NK cell-based immunotherapy in renal cell carcinoma. Cancers. (2020) 12:107848. doi: 10.3390/cancers12020316 PMC7072691 32013092

[54] Tietze JK. Tumorinfiltrierende T-Zellen und natürliche Killerzellen im Melanom. Die Dermatologie. (2022) 73:929–36. doi: 10.1007/s00105-022-05076-4 36401123

[55] Rezaeifard S Talei A Shariat M Erfani N. Tumor infiltrating NK cell (TINK) subsets and functional molecules in patients with breast cancer. Mol Immunol. (2021) 136:161–7. doi: 10.1016/j.molimm.2021.03.003 34171565

[56] Pockley AG Vaupel P Multhoff G. NK cell-based therapeutics for lung cancer. Expert Opin Biol Ther. (2020) 20:23–33. doi: 10.1080/14712598.2020.1688298 31714156

[57] Sordo-Bahamonde C Vitale M Lorenzo-Herrero S López-Soto A Gonzalez S. Mechanisms of resistance to NK cell immunotherapy. Cancers. (2020) 12:893. doi: 10.3390/cancers12040893 32272610 PMC7226138

[58] Kaur K Chen P-C Ko M-W Mei A Senjor E Malarkannan S. Sequential therapy with supercharged NK cells with either chemotherapy drug cisplatin or anti-PD-1 antibody decreases the tumor size and significantly enhances the NK function in Hu-BLT mice. Front Immunol. (2023) 14:2023. doi: 10.3389/fimmu.2023.1132807 PMC10183580 37197660

[59] McGowan E Lin Q Ma G Yin H Chen S Lin Y. PD-1 disrupted CAR-T cells in the treatment of solid tumors: Promises and challenges. Biomed Pharmacother. (2020) 121:109625. doi: 10.1016/j.biopha.2019.109625 31733578

[60] Rupp LJ Schumann K Roybal KT Gate RE Ye CJ Lim WA. CRISPR/Cas9-mediated PD-1 disruption enhances anti-tumor efficacy of human chimeric antigen receptor T cells. Sci Rep. (2017) 7:737. doi: 10.1038/s41598-017-00462-8 28389661 PMC5428439

[61] McDermott DF Lee J-L Bjarnason GA Larkin JMG Gafanov RA Kochenderfer MD. Open-label, single-arm phase II study of pembrolizumab monotherapy as first-line therapy in patients with advanced clear cell renal cell carcinoma. J Clin Oncol. (2021) 39:1020–8. doi: 10.1200/JCO.20.02363 PMC8078336 33529051

[62] Wu J Mishra HK Walcheck B. Role of ADAM17 as a regulatory checkpoint of CD16A in NK cells and as a potential target for cancer immunotherapy. J leukocyte Biol. (2019) 105:1297–303. doi: 10.1002/JLB.2MR1218-501R PMC6792391 30786043

[63] Liu E Marin D Banerjee P Macapinlac Homer A Thompson P Basar R. Use of CAR-transduced natural killer cells in CD19-positive lymphoid tumors. New Engl J Med. (2020) 382:545–53. doi: 10.1056/NEJMoa1910607 PMC7101242 32023374

[64] Capuano C Pighi C Battella S De Federicis D Galandrini R Palmieri G. Harnessing CD16-mediated NK cell functions to enhance therapeutic efficacy of tumor-targeting mAbs. Cancers. (2021) 13:2500. doi: 10.3390/cancers13102500 34065399 PMC8161310

[65] Neelapu SS Tummala S Kebriaei P Wierda W Gutierrez C Locke FL. Chimeric antigen receptor T-cell therapy — assessment and management of toxicities. Nat Rev Clin Oncol. (2018) 15:47–62. doi: 10.1038/nrclinonc.2017.148 28925994 PMC6733403

[66] Lee DW Gardner R Porter DL Louis CU Ahmed N Jensen M. Current concepts in the diagnosis and management of cytokine release syndrome. Blood J Am Soc Hematol. (2014) 124:188–95. doi: 10.1182/blood-2014-05-552729 PMC4093680 24876563

[67] Fraietta JA Nobles CL Sammons MA Lundh S Carty SA Reich TJ. Disruption of TET2 promotes the therapeutic efficacy of CD19-targeted T cells. Nature. (2018) 558:307–12. doi: 10.1038/s41586-018-0178-z PMC6320248 29849141

[68] Su-fern Seng M Soh SY Lim FL Hwang WYK Koh LP Chan E. Manufacturing characteristics and early outcomes of a tandem aCD22-aCD19 CAR-T therapy for childhood and adult relapsed/refractory acute precursor B lymphoblastic leukemia. Transplant Cell Ther. (2025) 31:S11. doi: 10.1016/j.jtct.2025.01.020

[69] Sterner RC Sterner RM. CAR-T cell therapy: current limitations and potential strategies. Blood Cancer J. (2021) 11:69. doi: 10.1038/s41408-021-00459-7 33824268 PMC8024391

[70] Pang Z Wang Z Li F Feng C Mu X. Current progress of CAR-NK therapy in cancer treatment. Cancers. (2022) 14:4318. doi: 10.3390/cancers14174318 36077853 PMC9454439

[71] Wrona E Borowiec M Potemski P. CAR-NK cells in the treatment of solid tumors. Int J Mol Sci. (2021) 22:5899. doi: 10.3390/ijms22115899 34072732 PMC8197981

[72] Lu H Zhao X Li Z Hu Y Wang H. From CAR-T cells to CAR-NK cells: a developing immunotherapy method for hematological Malignancies. Front Oncol. (2021) 11:720501. doi: 10.3389/fonc.2021.720501 34422667 PMC8377427

[73] Peng L Sferruzza G Yang L Zhou L Chen S. CAR-T and CAR-NK as cellular cancer immunotherapy for solid tumors. Cell Mol Immunol. (2024) 21:1089–108. doi: 10.1038/s41423-024-01207-0 PMC11442786 39134804

[74] Krug A Martinez-Turtos A Verhoeyen E. Importance of T, NK, CAR T and CAR NK cell metabolic fitness for effective anti-cancer therapy: A continuous learning process allowing the optimization of T, NK and CAR-based anti-cancer therapies. Cancers. (2022) 14:183. doi: 10.3390/cancers14010183 PMC8782435 35008348

[75] Keshavarz A Salehi A Khosravi S Shariati Y Nasrabadi N Kahrizi MS. Recent findings on chimeric antigen receptor (CAR)-engineered immune cell therapy in solid tumors and hematological Malignancies. Stem Cell Res Ther. (2022) 13:482. doi: 10.1186/s13287-022-03163-w 36153626 PMC9509604

[76] Balkhi S Zuccolotto G Di Spirito A Rosato A Mortara L. CAR-NK cell therapy: promise and challenges in solid tumors. Front Immunol. (2025) 16:2025. doi: 10.3389/fimmu.2025.1574742 PMC12009813 40260240

[77] Valeri A García-Ortiz A Castellano E Córdoba L Maroto-Martín E Encinas J. Overcoming tumor resistance mechanisms in CAR-NK cell therapy. Front Immunol. (2022) 13:953849. doi: 10.3389/fimmu.2022.953849 35990652 PMC9381932

[78] Kloess S Oberschmidt O Dahlke J Vu X-K Neudoerfl C Kloos A. Preclinical assessment of suitable natural killer cell sources for chimeric antigen receptor natural killer–based “Off-the-shelf” Acute myeloid leukemia immunotherapies. Hum Gene Ther. (2019) 30:381–401. doi: 10.1089/hum.2018.247 30734584

[79] Habib S Tariq SM Tariq M. Chimeric antigen receptor-natural killer cells: the future of cancer immunotherapy. Ochsner J. (2019) 19:186–7. doi: 10.31486/toj.19.0033 PMC6735593 31528126

[80] Franks SE Wolfson B Hodge JW. Natural born killers: NK cells in cancer therapy. Cancers. (2020) 12:2131. doi: 10.3390/cancers12082131 32751977 PMC7465121

[81] Zhang L Meng Y Feng X Han Z. CAR-NK cells for cancer immunotherapy: From bench to bedside. Biomarker Res. (2022) 10:1–19. doi: 10.1186/s40364-022-00364-6 PMC8932134 35303962

[82] Bashash D Zandi Z Kashani B Pourbagheri-Sigaroodi A Salari S Ghaffari SH. Resistance to immunotherapy in human Malignancies: Mechanisms, research progresses, challenges, and opportunities. J Cell Physiol. (2022) 237:346–72. doi: 10.1002/jcp.v237.1 34498289

[83] Dobosz P Stępień M Golke A Dzieciątkowski T. Challenges of the immunotherapy: perspectives and limitations of the immune checkpoint inhibitor treatment. Int J Mol Sci. (2022) 23:2847. doi: 10.3390/ijms23052847 35269988 PMC8910928

[84] Wei R Liu S Zhang S Min L Zhu S. Cellular and extracellular components in tumor microenvironment and their application in early diagnosis of cancers. Analytical Cell Pathol. (2020) 2020:6283796. doi: 10.1155/2020/6283796 PMC7199555 32377504

[85] Wu B Zhang B Li B Wu H Jiang M. Cold and hot tumors: from molecular mechanisms to targeted therapy. Signal Transduction Targeted Ther. (2024) 9:274. doi: 10.1038/s41392-024-01979-x PMC11491057 39420203

[86] McKean WB Moser JC Rimm D Hu-Lieskovan S. Biomarkers in precision cancer immunotherapy: promise and challenges. Am Soc Clin Oncol Educ Book. (2020) 40:e275–91. doi: 10.1200/EDBK_280571 32453632

[87] Gunaydin G. CAFs interacting with TAMs in tumor microenvironment to enhance tumorigenesis and immune evasion. Front Oncol. (2021) 11:668349. doi: 10.3389/fonc.2021.668349 34336660 PMC8317617

[88] Vesely MD Zhang T Chen L. Resistance mechanisms to anti-PD cancer immunotherapy. Annu Rev Immunol. (2022) 40:45–74. doi: 10.1146/annurev-immunol-070621-030155 35471840

[89] Guo X Wang G. Advances in research on immune escape mechanism of glioma. CNS Neurosci Ther. (2023) 29:1709–20. doi: 10.1111/cns.14217 PMC10324367 37088950

[90] Vitale I Shema E Loi S Galluzzi L. Intratumoral heterogeneity in cancer progression and response to immunotherapy. Nat Med. (2021) 27:212–24. doi: 10.1038/s41591-021-01233-9 33574607

[91] Tanaka M Lum L Ledezma-Soto C Hu K Superville D Adams Z. An immunosuppressive tumor population drives immunotherapy resistance of heterogeneous tumors. J Immunol. (2023) 210:230.04. doi: 10.4049/jimmunol.210.Supp.230.04

[92] Asiry S Kim G Filippou PS Sanchez LR Entenberg D Marks DK. The cancer cell dissemination machinery as an immunosuppressive niche: a new obstacle towards the era of cancer immunotherapy. Front Immunol. (2021) 12:654877. doi: 10.3389/fimmu.2021.654877 33927723 PMC8076861

[93] Jones B Koch AE Ahmed S. Pathological role of fractalkine/CX3CL1 in rheumatic diseases: a unique chemokine with multiple functions. Front Immunol. (2012) 2:82. doi: 10.3389/fimmu.2011.00082 22566871 PMC3341950

[94] Taylor BC Balko JM. Mechanisms of MHC-I downregulation and role in immunotherapy response. Front Immunol. (2022) 13:844866. doi: 10.3389/fimmu.2022.844866 35296095 PMC8920040

[95] Jia Q Wang A Yuan Y Zhu B Long H. Heterogeneity of the tumor immune microenvironment and its clinical relevance. Exp Hematol Oncol. (2022) 11:24. doi: 10.1186/s40164-022-00277-y 35461288 PMC9034473

[96] Sharma P Hu-Lieskovan S Wargo JA Ribas A. Primary, adaptive, and acquired resistance to cancer immunotherapy. Cell. (2017) 168:707–23. doi: 10.1016/j.cell.2017.01.017 PMC5391692 28187290

[97] Bai R Chen N Li L Du N Bai L Lv Z. Mechanisms of cancer resistance to immunotherapy. Front Oncol. (2020) 10:1290. doi: 10.3389/fonc.2020.01290 32850400 PMC7425302

[98] Shivani T Saleha S Rajiv P Vivek S. Unlocking the potential of immunomodulators as synergistic immune-based therapies in cancer. Discov Med. (2025) 37:411–32. doi: 10.24976/Discov.Med.202537194.35 40116091

[99] Prajapati S Yadav S. Revolutionizing glioblastoma immunotherapy conquering transport and biological challenges, innovating combinatorial approaches for unprecedented treatment success. Clin Cancer Drugs. (2024) 10:18. doi: 10.2174/012212697X332800241103174044

[100] Kim N Lee D-H Choi WS Yi E Kim H Kim JM. Harnessing NK cells for cancer immunotherapy: immune checkpoint receptors and chimeric antigen receptors. BMB Rep. (2021) 54:44. doi: 10.5483/BMBRep.2021.54.1.214 33298244 PMC7851441

[101] Fantini M Arlen PM Tsang KY. Potentiation of natural killer cells to overcome cancer resistance to NK cell-based therapy and to enhance antibody-based immunotherapy. Front Immunol. (2023) 14:1275904. doi: 10.3389/fimmu.2023.1275904 38077389 PMC10704476

[102] Yerragopu AK Vellapandian C. Chemoimmunotherapy with doxorubicin and caffeine combination enhanced ICD induction and T-cell infiltration in B16F10 melanoma tumors. J Biochem Mol Toxicol. (2023) 37:e23327. doi: 10.1002/jbt.23327 36807623

[103] Kang TH Mao C-P Lee SY Chen A Lee J-H Kim TW. Chemotherapy acts as an adjuvant to convert the tumor microenvironment into a highly permissive state for vaccination-induced antitumor immunity. Cancer Res. (2013) 73:2493–504. doi: 10.1158/0008-5472.CAN-12-4241 PMC3630272 23418322

[104] Bie N Yong T Wei Z Liang Q Zhang X Li S. Tumor-repopulating cell-derived microparticles elicit cascade amplification of chemotherapy-induced antitumor immunity to boost anti-PD-1 therapy. Signal Transduction Targeted Ther. (2023) 8:408. doi: 10.1038/s41392-023-01658-3 PMC10598206 37875473

[105] Behranvand N Nasri F Zolfaghari Emameh R Khani P Hosseini A Garssen J. Chemotherapy: a double-edged sword in cancer treatment. Cancer Immunol Immunother. (2022) 71:507–26. doi: 10.1007/s00262-021-03013-3 PMC10992618 34355266

[106] Nakajima S Mimura K Saito K Thar Min AK Endo E Yamada L. Neoadjuvant chemotherapy induces IL34 signaling and promotes chemoresistance via tumor-associated macrophage polarization in esophageal squamous cell carcinoma. Mol Cancer Res. (2021) 19:1085–95. doi: 10.1158/1541-7786.MCR-20-0917 33674443

[107] Gómez-Valenzuela F Escobar E Pérez-Tomás R Montecinos VP. The inflammatory profile of the tumor microenvironment, orchestrated by cyclooxygenase-2, promotes epithelial-mesenchymal transition. Front Oncol. (2021) 11. doi: 10.3389/fonc.2021.686792 PMC8222670 34178680

[108] Zingoni A Fionda C Borrelli C Cippitelli M Santoni A Soriani A. Natural killer cell response to chemotherapy-stressed cancer cells: role in tumor immunosurveillance. Front Immunol. (2017) 8. doi: 10.3389/fimmu.2017.01194 PMC5622151 28993779

[109] Schwermann M Huntington KE Carlsen L Zhou L Srinivasan P George A. Abstract 1843: Androgen signaling blockade enhances NK cell-mediated killing of prostate cancer cells (PC) and promotes NK cell tumor infiltration in *vivo* . Cancer Res. (2023) 83:1843–3. doi: 10.1158/1538-7445.AM2023-1843

[110] Garofalo C De Marco C Cristiani CM. NK cells in the tumor microenvironment as new potential players mediating chemotherapy effects in metastatic melanoma. Front Oncol. (2021) 11. doi: 10.3389/fonc.2021.754541 PMC8547654 34712615

[111] Kim R Kawai A Wakisaka M Funaoka Y Yasuda N Hidaka M. A potential role for peripheral natural killer cell activity induced by preoperative chemotherapy in breast cancer patients. Cancer Immunol Immunother. (2019) 68:577–85. doi: 10.1007/s00262-019-02305-z PMC11028034 30673828

[112] Huang W Chen J-J Xing R Zeng Y-C. Combination therapy: future directions of immunotherapy in small cell lung cancer. Trans Oncol. (2021) 14:100889. doi: 10.1016/j.tranon.2020.100889 PMC7567053 33065386

[113] Shimasaki N Jain A Campana D. NK cells for cancer immunotherapy. Nat Rev Drug Discov. (2020) 19:200–18. doi: 10.1038/s41573-019-0052-1 31907401

[114] Theelen WS Peulen HM Lalezari F van der Noort V De Vries JF Aerts JG. Effect of pembrolizumab after stereotactic body radiotherapy vs pembrolizumab alone on tumor response in patients with advanced non–small cell lung cancer: results of the PEMBRO-RT phase 2 randomized clinical trial. JAMA Oncol. (2019) 5:1276–82. doi: 10.1001/jamaoncol.2019.1478 PMC6624814 31294749

[115] Zhu S Wang Y Tang J Cao M. Radiotherapy induced immunogenic cell death by remodeling tumor immune microenvironment. Front Immunol. (2022) 13:1074477. doi: 10.3389/fimmu.2022.1074477 36532071 PMC9753984

[116] Wattenberg MM Fahim A Ahmed MM Hodge JW. Unlocking the combination: potentiation of radiation-induced antitumor responses with immunotherapy. Radiat Res. (2014) 182:126–38. doi: 10.1667/RR13374.1 PMC4128341 24960415

[117] Gong Y Klein Wolterink RG Wang J Bos GM Germeraad WT. Chimeric antigen receptor natural killer (CAR-NK) cell design and engineering for cancer therapy. J Hematol Oncol. (2021) 14:1–35. doi: 10.1186/s13045-021-01083-5 33933160 PMC8088725

[118] Kennedy PR Vallera DA Ettestad B Hallstrom C Kodal B Todhunter DA. A tri-specific killer engager against mesothelin targets NK cells towards lung cancer. Front Immunol. (2023) 14:1060905. doi: 10.3389/fimmu.2023.1060905 36911670 PMC9992642

[119] Rafei H Daher M Rezvani K. Chimeric antigen receptor (CAR) natural killer (NK)-cell therapy: leveraging the power of innate immunity. Br J haematol. (2021) 193:216–30. doi: 10.1111/bjh.v193.2 PMC9942693 33216984

[120] Romee R Leong JW Fehniger TA. Utilizing cytokines to function-enable human NK cells for the immunotherapy of cancer. Scientifica. (2014) 2014:205796. doi: 10.1155/2014/205796 25054077 PMC4099226

[121] Cheng M Ma J Chen Y Zhang J Zhao W Zhang J. Establishment, characterization, and successful adaptive therapy against human tumors of NKG cell, a new human NK cell line. Cell Transplant. (2011) 20:1731–46. doi: 10.3727/096368911X580536 21669033

[122] Vahedi F Nham T Poznanski SM Chew MV Shenouda MM Lee D. Ex vivo expanded human NK cells survive and proliferate in humanized mice with autologous human immune cells. Sci Rep. (2017) 7:12083. doi: 10.1038/s41598-017-12223-8 28935883 PMC5608690

[123] Eguizabal C Zenarruzabeitia O Monge J Santos S Vesga MA Maruri N. Natural killer cells for cancer immunotherapy: pluripotent stem cells-derived NK cells as an immunotherapeutic perspective. Front Immunol. (2014) 5:439. doi: 10.3389/fimmu.2014.00439 25309538 PMC4164009

[124] Khawar MB Afzal A Si Y Sun H. Steering the course of CAR T cell therapy with lipid nanoparticles. J Nanobiotechnol. (2024) 22:380. doi: 10.1186/s12951-024-02630-1 PMC11212433 38943167

[125] Basar R Daher MD Uprety N Ensley E Nunez Cortes AK Acharya S. DAP10 co-stimulation imparts memory-like features to CD5 targeting cord blood derived CAR-NK cells. Blood. (2023) 142:2089–9. doi: 10.1182/blood-2023-187665

[126] Wilson JL Charo J Martín-Fontecha A Dellabona P Casorati G Chambers BJ. NK cell triggering by the human costimulatory molecules CD80 and CD86. J Immunol. (1999) 163:4207–12. doi: 10.4049/jimmunol.163.8.4207 10510357

[127] Marotel M Hasim MS Hagerman A Ardolino M. The two-faces of NK cells in oncolytic virotherapy. Cytokine Growth Factor Rev. (2020) 56:59–68. doi: 10.1016/j.cytogfr.2020.06.005 32586674

[128] Albinger N Bexte T Buchinger L Wendel P Al-Ajami A Gessner A. CRISPR/Cas9 gene editing of immune checkpoint receptor NKG2A improves the efficacy of primary CD33-CAR-NK cells against AML. Blood. (2022) 140:4558–9. doi: 10.1182/blood-2022-169758

[129] Jetani H Romero P Kunert A Franciszkiewicz K Vlerick D Nowicka A. 272 PD-L1 CAR engineered K-NK cells to target PD-L1+ or PD-L1-tumors. BMJ Specialist Journals. (2023) 11:A312–A312. doi: 10.1136/jitc-2023-SITC2023.0272

[130] Khan M Zhao Z Arooj S Fu Y Liao G. Soluble PD-1: predictive, prognostic, and therapeutic value for cancer immunotherapy. Front Immunol. (2020) 11:587460. doi: 10.3389/fimmu.2020.587460 33329567 PMC7710690

[131] Dysthe M Navin I Baumgartner C Fetzko S Rooney C Parihar R. 328 Promoting NK survival and function within the tumor microenvironment. BMJ Specialist Journals. (2022) 10:A345. doi: 10.1136/jitc-2022-SITC2022.0328

[132] Liu Z Li J Zhao F Ren D Li Z Chen Y. Long-term survival after neoadjuvant therapy for triple-negative breast cancer under different treatment regimens: a systematic review and network meta-analysis. BMC Cancer. (2024) 24:440. doi: 10.1186/s12885-024-12222-9 38594636 PMC11005293

[133] Robbins GM Wang M Pomeroy EJ Moriarity BS. Nonviral genome engineering of natural killer cells. Stem Cell Res Ther. (2021) 12:350. doi: 10.1186/s13287-021-02406-6 34134774 PMC8207670

[134] Wu X Matosevic S. Gene-edited and CAR-NK cells: Opportunities and challenges with engineering of NK cells for immunotherapy. Mol Therapy-Oncolytics. (2022) 27:224–38. doi: 10.1016/j.omto.2022.10.011 PMC9676278 36420307

[135] Feigl FF Stahringer A Peindl M Dandekar G Koehl U Fricke S. Efficient redirection of NK cells by genetic modification with chemokine receptors CCR4 and CCR2B. Int J Mol Sci. (2023) 24:3129. doi: 10.3390/ijms24043129 36834542 PMC9967507

[136] Levy ER Carlsten M Childs RW. mRNA transfection to improve NK cell homing to tumors. Natural killer cells: methods and protocols. Somanchi SS, editor. New York, NY: Springer New York (2016) p. 231–40. 10.1007/978-1-4939-3684-7_19 27177670

[137] Carlsten M Levy E Karambelkar A Li L Reger R Berg M. Efficient mRNA-Based Genetic Engineering of Human NK Cells with High-Affinity CD16 and CCR7 Augments Rituximab-Induced ADCC against Lymphoma and Targets NK Cell Migration toward the Lymph Node-Associated Chemokine CCL19. Front Immunol. (2016) 7. doi: 10.3389/fimmu.2016.00105 PMC4801851 27047492

[138] Abdelbary R Ragheb M El Sobky SA El-Badri N Aboud N Tawheed A. MiR-216a-3p inhibits the cytotoxicity of primary natural killer cells. Front Oncol. (2025) 14:1523068. doi: 10.3389/fonc.2024.1523068 39906666 PMC11790671

[139] Jorgensen P Edgington NP Schneider BL Rupeš I Tyers M Futcher B. The size of the nucleus increases as yeast cells grow. Mol Biol Cell. (2007) 18:3523–32. doi: 10.1091/mbc.e06-10-0973 PMC1951755 17596521

[140] Moradian H Roch T Lendlein A Gossen M. mRNA transfection-induced activation of primary human monocytes and macrophages: dependence on carrier system and nucleotide modification. Sci Rep. (2020) 10:4181. doi: 10.1038/s41598-020-60506-4 32144280 PMC7060354

[141] Stadelmann C Di Francescantonio S Marg A Müthel S Spuler S Escobar H. mRNA-mediated delivery of gene editing tools to human primary muscle stem cells. Mol Therapy-Nucleic Acids. (2022) 28:47–57. doi: 10.1016/j.omtn.2022.02.016 PMC8931293 35356683

[142] Asad S Jacobsen A-C Teleki A. Inorganic nanoparticles for oral drug delivery: opportunities, barriers, and future perspectives. Curr Opin Chem Eng. (2022) 38:100869. doi: 10.1016/j.coche.2022.100869

[143] Mohseni M Connell JJ Payne C Patrick PS Baker R Yu Y. Scalable magnet geometries enhance tumour targeting of magnetic nano-carriers. Materials Design. (2020) 191:108610. doi: 10.1016/j.matdes.2020.108610

[144] Tsai H-C Pietrobon V Peng M Wang S Zhao L Marincola FM. Current strategies employed in the manipulation of gene expression for clinical purposes. J Trans Med. (2022) 20:535. doi: 10.1186/s12967-022-03747-3 PMC9673226 36401279

[145] VandenDriessche T Ivics Z Izsvák Z Chuah MKL. Emerging potential of transposons for gene therapy and generation of induced pluripotent stem cells. Blood. (2009) 114:1461–8. doi: 10.1182/blood-2009-04-210427 19471016

[146] Ivics Z Izsvák Z. The expanding universe of transposon technologies for gene and cell engineering. Mobile DNA. (2010) 1:25. doi: 10.1186/1759-8753-1-25 21138556 PMC3016246

[147] Tipanee J VandenDriessche T Chuah MK. Transposons: moving forward from preclinical studies to clinical trials. Hum Gene Ther. (2017) 28:1087–104. doi: 10.1089/hum.2017.128 28920716

[148] Hu LF Li YX Wang JZ Zhao YT Wang Y. Controlling CRISPR-Cas9 by guide RNA engineering. Wiley Interdiscip Reviews: RNA. (2023) 14:e1731. doi: 10.1002/wrna.v14.1 35393779

[149] Chen S Zhu H Jounaidi Y. Comprehensive snapshots of natural killer cells functions, signaling, molecular mechanisms and clinical utilization. Signal Transduction Targeted Ther. (2024) 9:302. doi: 10.1038/s41392-024-02005-w PMC11544004 39511139

[150] Mezu-Ndubuisi OJ Maheshwari A. The role of integrins in inflammation and angiogenesis. Pediatr Res. (2021) 89:1619–26. doi: 10.1038/s41390-020-01177-9 PMC8249239 33027803

[151] Shannon MJ Mace EM. Natural killer cell integrins and their functions in tissue residency. Front Immunol. (2021) 12. doi: 10.3389/fimmu.2021.647358 PMC7987804 33777044

[152] Freud AG Mundy-Bosse BL Yu J Caligiuri MA. The broad spectrum of human natural killer cell diversity. Immunity. (2017) 47:820–33. doi: 10.1016/j.immuni.2017.10.008 PMC5728700 29166586

[153] Rubio G Ferez X Mora A Galvez J Chicano A Garcia-Peñarrubia P. β1 Integrin triggering affects leukemic cell line sensitivity to natural killer cells. Cancer Immunol Immunother. (2002) 51:130–8. doi: 10.1007/s00262-002-0264-8 PMC11034235 11941451

[154] Bernardini G Sciumè G Santoni A. Differential chemotactic receptor requirements for NK cell subset trafficking into bone marrow. Front Immunol. (2013) 4:12. doi: 10.3389/fimmu.2013.00012 23386850 PMC3558687

[155] Nersesian S Schwartz SL Grantham SR MacLean LK Lee SN Pugh-Toole M. NK cell infiltration is associated with improved overall survival in solid cancers: A systematic review and meta-analysis. Trans Oncol. (2021) 14:100930. doi: 10.1016/j.tranon.2020.100930 PMC7670197 33186888

[156] Portale F Di Mitri D. NK cells in cancer: mechanisms of dysfunction and therapeutic potential. Int J Mol Sci. (2023) 24:9521. doi: 10.3390/ijms24119521 37298470 PMC10253405

[157] Bald T Krummel MF Smyth MJ Barry KC. The NK cell–cancer cycle: advances and new challenges in NK cell–based immunotherapies. Nat Immunol. (2020) 21:835–47. doi: 10.1038/s41590-020-0728-z PMC8406687 32690952

[158] Hu Z Xu X Wei H. The adverse impact of tumor microenvironment on NK-cell. Front Immunol. (2021) 12. doi: 10.3389/fimmu.2021.633361 PMC8226132 34177887

[159] Nakazawa T Morimoto T Maeoka R Matsuda R Nakamura M Nishimura F. CIS deletion by CRISPR/Cas9 enhances human primary natural killer cell functions against allogeneic glioblastoma. J Exp Clin Cancer Res. (2023) 42:205. doi: 10.1186/s13046-023-02770-6 37563692 PMC10413513

[160] Balke-Want H Keerthi V Gkitsas N Mancini AG Kurgan GL Fowler C. Homology-independent targeted insertion (HITI) enables guided CAR knock-in and efficient clinical scale CAR-T cell manufacturing. Mol Cancer. (2023) 22:100. doi: 10.1186/s12943-023-01799-7 37365642 PMC10291796

[161] Daher M Basar R Gokdemir E Baran N Uprety N Nunez Cortes AK. Targeting a cytokine checkpoint enhances the fitness of armored cord blood CAR-NK cells. Blood J Am Soc Hematol. (2021) 137:624–36. doi: 10.1182/blood.2020007748 PMC7869185 32902645

[162] Yang M Zeng C Li P Qian L Ding B Huang L. Impact of CXCR4 and CXCR7 knockout by CRISPR/Cas9 on the function of triple-negative breast cancer cells. OncoTargets Ther. (2019) 12:3849–58. doi: 10.2147/OTT.S195661 PMC6527053 31190884

[163] Tian Y Li Y Shao Y Zhang Y. Gene modification strategies for next-generation CAR T cells against solid cancers. J Hematol Oncol. (2020) 13:54. doi: 10.1186/s13045-020-00890-6 32423475 PMC7236186

[164] Adusumilli PS Cherkassky L Villena-Vargas J Colovos C Servais E Plotkin J. Regional delivery of mesothelin-targeted CAR T cell therapy generates potent and long-lasting CD4-dependent tumor immunity. Sci Trans Med. (2014) 6:261ra151–261ra151. doi: 10.1126/scitranslmed.3010162 PMC4373413 25378643

[165] Zhang C Liu Y. Targeting NK cell checkpoint receptors or molecules for cancer immunotherapy. Front Immunol. (2020) 11:1295. doi: 10.3389/fimmu.2020.01295 32714324 PMC7344328

[166] Heyman B Yang Y. Chimeric antigen receptor T cell therapy for solid tumors: current status, obstacles and future strategies. Cancers. (2019) 11:191. doi: 10.3390/cancers11020191 30736355 PMC6407020

[167] Nayyar G Chu Y Cairo MS. Overcoming resistance to natural killer cell based immunotherapies for solid tumors. Front Oncol. (2019) 9:51. doi: 10.3389/fonc.2019.00051 30805309 PMC6378304

[168] Marofi F Saleh MM Rahman HS Suksatan W Al-Gazally ME Abdelbasset WK. CAR-engineered NK cells; a promising therapeutic option for treatment of hematological Malignancies. Stem Cell Res Ther. (2021) 12:374. doi: 10.1186/s13287-021-02462-y 34215336 PMC8252313

[169] Daver N Alotaibi AS Bücklein V Subklewe M. T-cell-based immunotherapy of acute myeloid leukemia: current concepts and future developments. Leukemia. (2021) 35:1843–63. doi: 10.1038/s41375-021-01253-x PMC8257483 33953290

